# Tau protein profiling in tauopathies: a human brain study

**DOI:** 10.1186/s13024-024-00741-9

**Published:** 2024-07-19

**Authors:** Juan Lantero-Rodriguez, Elena Camporesi, Laia Montoliu-Gaya, Johan Gobom, Diana Piotrowska, Maria Olsson, Irena Matečko Burmann, Bruno Becker, Ann Brinkmalm, Björn M. Burmann, Michael Perkinton, Nicholas J. Ashton, Nick C. Fox, Tammaryn Lashley, Henrik Zetterberg, Kaj Blennow, Gunnar Brinkmalm

**Affiliations:** 1 Department of Psychiatry & Neurochemistry, Institute of Neuroscience and Physiology, The Sahlgrenska Academy at the University of Gothenburg, Mölndal, Sweden; 2 Clinical Neurochemistry Laboratory, Sahlgrenska University Hospital, Mölndal, Sweden; 3 Department of Chemistry and Molecular Biology, University of Gothenburg, Gothenburg, Sweden; 4 Wallenberg Centre for Molecular and Translational Medicine, University of Gothenburg, Gothenburg, Sweden; 5 AstraZeneca Neuroscience Innovative Medicines, MedImmune Ltd, Cambridge, CB21 6GH UK; 6 Centre for Age-Related Medicine, Stavanger University Hospital, Stavanger, Norway; 7 Department of Old Age Psychiatry, Maurice Wohl Clinical Neuroscience Institute, King’s College London, London, Maurice UK; 8 NIHR Biomedical Research Centre for Mental Health & Biomedical Research Unit for Dementia at South London & Maudsley NHS Foundation, London, UK; 9 Department of Neurodegenerative Disease, Queen Square Institute of Neurology, University College London, London, UK; 10 UK Dementia Research Institute, University College London, London, UK; 11 Hong Kong Center for Neurodegenerative Diseases, Hong Kong, China; 12 Wisconsin Alzheimer’s Disease Research Center, University of Wisconsin School of Medicine and Public Health, University of Wisconsin-Madison, Madison, WI USA

**Keywords:** Tau, Tauopathies, Mass spectrometry, Phosphorylation, Brain

## Abstract

Abnormal accumulation of misfolded and hyperphosphorylated tau protein in brain is the defining feature of several neurodegenerative diseases called tauopathies, including Alzheimer’s disease (AD). In AD, this pathological change is reflected by highly specific cerebrospinal fluid (CSF) tau biomarkers, including both phosphorylated and non-phosphorylated variants. Interestingly, despite tau pathology being at the core of all tauopathies, CSF tau biomarkers remain unchanged in certain tauopathies, e.g., progressive supranuclear palsy (PSP), Pick’s disease (PiD), and corticobasal neurodegeneration (CBD). To better understand commonalities and differences between tauopathies, we report a multiplex assay combining immunoprecipitation and high-resolution mass spectrometry capable of detecting and quantifying peptides from different tau protein isoforms as well as non-phosphorylated and phosphorylated peptides, including those carrying multiple phosphorylations. We investigated the tau proteoforms in soluble and insoluble fractions of brain tissue from subjects with autopsy-confirmed tauopathies, including sporadic AD (*n* = 10), PSP (*n* = 11), PiD (*n* = 10), and CBD (*n* = 10), and controls (*n* = 10). Our results demonstrate that non-phosphorylated tau profiles differ across tauopathies, generally showing high abundance of microtubule-binding region (MTBR)-containing peptides in insoluble protein fractions compared with controls; the AD group showed 12–72 times higher levels of MTBR-containing aggregates. Quantification of tau isoforms showed the 3R being more abundant in PiD and the 4R isoform being more abundant in CBD and PSP in the insoluble fraction. Twenty-three different phosphorylated peptides were quantified. Most phosphorylated peptides were measurable in all investigated tauopathies. All phosphorylated peptides were significantly increased in AD insoluble fraction. However, doubly and triply phosphorylated peptides were significantly increased in AD even in the soluble fraction. Results were replicated using a validation cohort comprising AD (*n* = 10), CBD (*n* = 10), and controls (*n* = 10). Our study demonstrates that abnormal levels of phosphorylation and aggregation do indeed occur in non-AD tauopathies, however, both appear pronouncedly increased in AD, becoming a distinctive characteristic of AD pathology.

**Supplementary Information:**

The online version contains supplementary material available at 10.1186/s13024-024-00741-9.

## Introduction

The term tauopathy refers to a large and disparate group of neurodegenerative diseases in which the tau protein is a characteristic and defining feature of the underlying pathology [1]. Despite having tau pathology in common, these diseases are heterogeneous, both clinically and pathologically [2, 3]. Tauopathies can be classified based on the contribution of tau to the overall pathology into primary tauopathies, if tau protein is believed to be the main driving cause of the disease, and secondary tauopathies, in which tau pathology is downstream of other neurodegenerative disease processes [4]. Examples of primary tauopathies include progressive supranuclear palsy (PSP), corticobasal degeneration (CBD), and Pick’s disease (PiD) [5], while secondary tauopathies are typically exemplified by Alzheimer’s disease (AD), the most prevalent cause of dementia, in which amyloid-β (Aβ) plaques are the primary cause [6]. At the microscopic level, histopathological examination of post-mortem brain tissue reveals that tauopathies differ from one another in terms of composition and morphology of tau aggregates, cell types affected, and the spatiotemporal involvement or spreading of pathological tau inclusions [7]. For example, AD is neuropathologically characterized, together with extracellular Aβ plaques, by intraneuronal accumulations of highly phosphorylated fibrillary tau, typically forming neurofibrillary tangles (NFTs) and neuropil threads, as well as dystrophic neurites surrounding the amyloid plaques [8–11]. Tau aggregates in primary tauopathies differ in morphology and composition from those found in AD [12, 13], and interestingly, tau deposits in primary tauopathies can be observed beyond neurons in cells such as astrocytes and oligodendrocytes (unlike in AD, where mainly neurons are affected) [3]. Moreover, different tau aggregates across tauopathies show different predominance of tau protein containing 3 or 4 microtubule-binding repeats (MTBR), respectively referred to as 3R and 4R isoforms [14]. Thus, following this criterion, tauopathies can also be classified into 3R-tauopathies e.g., PiD), 4R-tauopathies (e.g., PSP and CBD), or mixed 3R/4R tauopathies (e.g., AD). This pathological heterogeneity is also reflected in a wide range of clinical presentations that, together with the frequent presence of concomitant pathologies, make the clinical diagnosis of tauopathies challenging. Thus, post-mortem brain examination is still the gold standard for a definitive diagnosis for all tauopathies.

Hence, the study of the tau proteoforms in human brain may be critical to understand how a heterogeneous variety of pathological constructs can manifest from abnormalities in a single protein. For example, recent cryo-electron microscopy studies [15–18] have shown that different tauopathies appear to be characterized by different tau conformers. A potential explanation for this diversity is the dysregulation of post-translational modifications (PTMs) [19]. Abnormal tau phosphorylation and truncation have traditionally been described as central to tau pathology, as these appear to alter the ability of tau to exert its canonical function of stabilizing axonal microtubules [20, 21]. Tau phosphorylation and proteolytic processing emerge as the most widely studied PTMs due to the early discovery of highly phosphorylated and truncated tau as the main component of NFTs [22, 23]. In AD, tau phosphorylation and truncation translate from the brain into cerebrospinal fluid (CSF), where increased levels of both total tau (t-tau) and phosphorylated forms (p-tau) have been used as specific biomarkers of neurodegeneration and AD, respectively [8, 24, 25]. The development of ultrasensitive platforms now allows these tau species to be detected also in blood [26–28]. However, phosphorylated tau aggregates are commonly found in brain tissue of non-AD tauopathies [7, 29]. Despite this, in non-AD tauopathies, tau fluid biomarkers remain largely unchanged in comparison to healthy controls and are thus of very limited diagnostic use [30–33]. Thus, despite tau pathology being at the very core of all non-AD tauopathies, these disorders are limited to markers of global neurodegeneration (e.g., neurofilament light) [34] as no disease-specific biomarkers are currently available. Whilst a combination of detailed clinical and neuroimaging assessments together with normal core CSF AD biomarkers values (e.g., Aβ42/40, p-tau, and t-tau) is indicative of a non-AD type dementia, making an accurate clinical diagnosis of secondary tauopathies during life remaining a major challenge.

The large number of potential phosphorylation sites on tau protein makes understanding their individual contribution to aggregation and pathology, and thus disease development, a difficult task. To address this challenge, mass spectrometry (MS) is a very useful tool to map phosphorylated sites of tau in different tauopathies [35–38]. Indeed, several recent MS studies investigated numerous tau PTMs in brains affected by different tauopathies [39–43]. However, most studies investigated AD only and were mainly focused on either soluble or insoluble protein brain extracts. While there is a strong association between tau aggregates and neurodegeneration [44, 45] exemplified by the spatio-temporal association between NFT deposition, atrophy, and cognitive deficits in AD [46, 47], several studies have indicated that this type of tau deposits might not represent toxic entities. Instead, intermediate tau species such as tau oligomers have been suggested to be responsible for neurotoxicity and spreading of tau pathology [48–52]. Thus, while brain protein insoluble in aqueous buffers without added solubilizing agents such as detergents can be more reflective of pathological tau inclusions, soluble tau including tau fragments, full length, and small oligomeric species might characterise the interphase between tau detachment from microtubules and tau aggregation, and hence important to target. Furthermore, investigation of soluble fractions can provide valuable insights into tau species with biomarker potential, as previously reported [53]. Therefore, studying tau protein in both soluble and insoluble brain extracts from the same samples is of great relevance to understand tau pathophysiology.

The aim of this study was to characterise the commonalities and differences of the tau protein in post-mortem-confirmed brain tissue of AD, PSP, CBD, PiD, and control cases in both soluble and insoluble brain homogenates. In order to do this, we developed a multiplex assay combining liquid chromatography and high-resolution MS capable of quantifying non-phosphorylated, isoform-specific, as well as singly, doubly, and triply phosphorylated tau peptides in soluble and insoluble brain protein extracts.

## Materials and methods

**Fig. 1:**
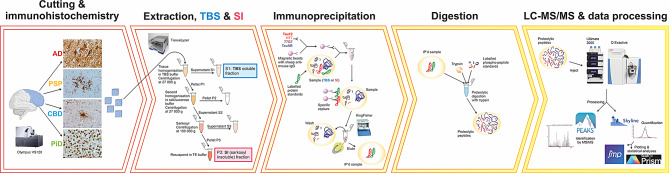
Overview of the sample preparation and analysis workflow

An overview of the sample preparation and analysis workflow is shown in Fig. 1. Neuropathologically confirmed brain tissues were selected for homogenisation and sequential separation into soluble and insoluble fractions. Fractions were then immunoprecipitated and the eluates analysed by liquid chromatography (LC)-MS.

### Post-mortem brain tissue

Post-mortem tissue from human brain donors was provided by the Queen Square Brain Bank for Neurological Disorders (QSBB), Department of Clinical and Movement Neurosciences, University College London (UCL) Queen Square Institute of Neurology. Frontal cortex was sampled from two cohorts; Cohort 1 consisting of control (*n* = 10), sporadic AD (*n* = 10), PSP (*n* = 11), CBD (*n* = 10), and PiD cases (*n* = 10) and Cohort 2 consisting of control (*n* = 10), sporadic AD (*n* = 10), and CBD cases (*n* = 10). The tissue was sampled from Broadman area 9 and was flash frozen on -80 °C brass plates when donated to QSBB and then stored at -80 °C. General demographics are summarised in Table 1 and full demographics are given in Suppl. Table 1. The neuropathological assessment of the AD cases fulfilled the 2012 National Institute on Aging and the Alzheimer’s Association (NIA-AA) guidelines [54] and the clinical evaluation met the National Institute of Neurological and Communicative Disorders and Stroke (NINCDS) criteria for probable AD [55]. Neuropathological diagnoses for PSP, CBD, and PiD were made according to their corresponding diagnostic criteria [56]. Control samples used in the study were cases with no neurological symptoms documented during life. Human brain tissues were used in accordance with the Helsinki declaration and the regional ethics committees at UCL and the University of Gothenburg.

**Table 1:** Brain samples demographics. For age at death and post-mortem delay data are presented as mean and (standard deviation)

		Brain region	Female (%)	Age at death (years)	Post-mortem delay, (hours)	Braak stages 0/I-II/III-IV/V-VI	Thal phases 0/1–2/3/4–5	CAA 0/1/2/3
Cohort 1	Control (*n* = 10)	FGM	6 (60%)	78.7 (17.7)	77.6 (42.8)	3/7/0/0	4/4/1/1	8/2/0/0
	AD (*n* = 10)	FGM	4 (40%)	69.4 (8.1)	53.8 (28.9)	0/0/0/10	0/0/0/10	1/1/1/7
	PSP (*n* = 11)	FGM	5 (45%)	79.3 (9.0)	47.9 (21.6)	na	na	na
	CBD (*n* = 10)	FGM	3 (30%)	68.6 (4.9)	73.0 (25.1)	na	na	na
	PiD (*n* = 10)	FGM	1 (10%)	69.8 (4.8)	67.4 (27.3)	na	na	na
Cohort 2	Control (*n* = 10)	FGM	2 (20%)	78.0 (7.0)	56.0 (30.6)	2/4/4/0	2/3/4/1	6/1/1/2
	AD (*n* = 10)	FGM	6 (60%)	75.8 (6.7)	58.6 (23.1)	0/0/0/10	0/0/0/10	0/2/2/6
	CBD (*n* = 10)	FGM	6 (60%)	71.0 (7.2)	78.0 (28.9)	3/4/2/0	4/2/0/5	6/2/1/0

FGM = frontal grey matter, CAA = cerebral amyloid angiopathy, AD = (sporadic) Alzheimer’s diseases, PSP = progressive sopranuclear palsy, CBD = corticobasal neurodegeneration, PiD = Pick’s disease, na = not analyzed

### Tissue homogenization and protein extraction

Brain tissue processing and protein extraction were performed as previously reported [57], ultimately generating two distinct fractions from each participant/case: tris(hydroxymethyl)aminomethane (tris)-buffered saline (TBS, S1) soluble, containing tau soluble forms and small oligomers, and sarkosyl-insoluble (SI, P3) fraction, containing higher molecular mass tau aggregates. Extraction of TBS-soluble fractions (S1) from brain homogenates is described in detail in [58]. The remaining pellets (P1) were further homogenized using 5 × volume/tissue mass of high salt–high sucrose buffer (10 mM Tris–HCl, 0.8 M NaCl, 10% sucrose, 1 mM ethylene glycol-bis(β-aminoethyl ether)-*N, N,N’,N’*-tetraacetic acid (EGTA), Complete Protease inhibitor Cocktail, Roche Diagnostic GmbH, pH 7.4). Homogenates were centrifuged at 27,000 × g for 20 min at 4 °C, after which supernatants (S2) and pellets (P2) were collected. For analysis, pellet P2 was reconstituted in 80% formic acid and shaking for 1 h followed by addition of PBS to obtain a pH suitable for IP, see Sect. Tau immunoprecipitation below. Sarkosyl detergent was added to the supernatants (S2) to a final concentration of 1%. Supernatants were then incubated with shaking for 1 h at 37 °C using a VorTemp 56 orbital shaker (Labnet). This was followed by an ultracentrifugation at 150,000 × g for 1 h at 4 °C. Resulting supernatant (S3) was collected and the pellet (P3) was resuspended in 0.5 × volume/tissue mass of tris-ethylenediaminetetraacetic acid (EDTA) buffer (10 mM Tris–HCl, 1 mM EDTA, pH 8.0). Total protein determination in TBS-soluble and SI fractions was determined using DC Protein Assay kit (Bio-Rad). Finally, all brain fractions were aliquoted and stored at -80 °C.

Initially all four obtained fractions (Fig. 1) were investigated for pooled control and AD brains. While fractions S1 (TBS) and P3 (SI) contained substantial amounts of tau, fractions P2 and S3 contained very little tau (data not shown). Therefore, all further analyses were carried out for TBS and SI fractions only.

Western blotting was performed to investigate aggregated tau in the TBS and SI fractions for controls, AD, and CBD; the sample amount added per lane were 5 µg and 1 µg, respectively. Blotting was performed using PHF-1 mAb detecting tau phosphorylated at Ser396/Ser404 (courtesy of Dr. Peter Davies). Samples were reduced in electrophoresis sample buffer containing 2.5% β-mercaptoethanol (v/v) at 60 °C for 15 min. Suppl. Figure 1 shows differences in tau forms contained in the TBS and SI fractions. PHF-1 positive signals appeared mostly as a smear of bands above 38 kDa up to the start of the blot (~ 250 kDa). The AD group exhibited the highest signal, followed by CBD, while the control group had weak signal. The signal was stronger in the SI fraction for all groups and particularly for AD, but also for CBD there was a clear sign of aggregated tau forms, corresponding to the black smear at higher MW positions (Suppl. Figure 1b). For AD the aggregated forms were visible also in the TBS fraction (Suppl. Figure 1a).

### Isotope-labelled standards

Two types of stable isotope-labelled standards were used; full length tau uniformly labelled [*U*-^15^N] and phosphorylated peptides amino acid specific labelled at lysine [^13^C,^15^N-Lys] or arginine residues [^13^C,^15^N-Arg].

#### Protein standards

The advantage of using protein standards is that they can be introduced early in the sample processing workflow, i.e., prior to immunoprecipitation (IP) and proteolytic digestion. central nervous system-specific tau consists of six different isoforms, see Suppl. Figure 2. However, the N and R inserts are well separated from each other in the primary structure so no tryptic peptides will contain both N and R inserts. Therefore, since the samples were to be digested with trypsin, three isoforms were sufficient to cover all peptides reflecting isoforms, so a mixture of 0N3R, 1N4R, and 2N4R isoforms was selected covering all three N as well as both R isoforms. [*U*-^15^N]-Labelled protein standards were produced in-house and described in Suppl. Information 1 and 2. The protein standards were titrated against a pool of TBS brain extract so that equal amounts (nominally 133 fmol) of each of the three isoforms could be added to the samples (described in detail in Suppl. Information 3).

#### Phosphorylated peptide standards

There is no straightforward way to generate protein standards with suitable phosphorylation profiles, therefore, phosphorylated peptide standards had to be utilised. Phosphate groups affect the ability of trypsin to cleave, so frequently the phosphorylated peptides will contain multiple lysines and/or arginines. The phospho-peptides selected for quantification were therefore not based on theoretical *in silico* digestion but on empirical data from TBS soluble brain extracts from both controls and AD patients. In most cases, peptides cannot be added before IP since they lack the antibody epitope, but they can be designed to mimic the proteolytic cleavage (at least to some extent). Therefore, we chose to use peptides that extended at least five amino acids both N- and C-terminally of the phospho-peptides selected for quantification. The amino acid selected for stable isotope labelling (with both ^13^C and ^15^N) was the C-terminal amino acid (either [^13^C,^15^N-Lys] or [^13^C,^15^N-Arg]) in each resulting tryptic phospho-peptide. Table 2 contains a list of the included peptides. All isotope labelled peptides were of “HeavyPeptide AQUA Basic” grade from Thermo Fisher Scientific. The labelled phospho-peptides were diluted, mixed, and 100 fmol of each were added to each sample after IP but before digestion with trypsin.

**Table 2:** Phosphorylated peptides quantified

Measured peptide	Extended-peptide standard^a^	Phosphate locations	Extended peptide sequence^b^
175-190-p1	170-195-p181	T181	RIPAKTPPAPK[T]PPSSGEPP(K)SGDRS
195-209-p1^c^	190-214-p202	S202	KSGDRSGYSSPG[S]PGTPGS(R)SRTPS
	190-214-p205	T205	KSGDRSGYSSPGSPG[T]PGS(R)SRTPS
195-209-p2	190-214-p202 + 205	S202 + T205	KSGDRSGYSSPG[S]PG[T]PGS(R)SRTPS
210-224-p1	205-229-p217	T217	TPGSRSRTPSLP[T]PPTREP(K)KVAVV
210-224-p2	205-229-p212 + 217	T212 + T217	TPGSRSR[T]PSLP[T]PPTREP(K)KVAVV
210-224-p3^d^	Same as for 210-224-p2		
212-224-p1^e^	205-229-p217	T217	TPGSRSRTPSLP[T]PPTREP(K)KVAVV
225-240-p1	220-245-p231	T231	TREPKKVAVVR[T]PPKSPSSA(K)SRLQT
225-240-p2^c^	220-245-p231 + 235	T231 + S235	TREPKKVAVVR[T]PPK[S]PSSA(K)SRLQT
	220-245-p231 + 237	T231 + S237	TREPKKVAVVR[T]PPKSP[S]SA(K)SRLQT
	220-245-p231 + 238	T231 + S238	TREPKKVAVVR[T]PPKSPS[S]A(K)SRLQT
225-240-p3^c^	220-245-p231 + 235 + 237	T231 + S235 + S237	TREPKKVAVVR[T]PPK[S]P[S]SA(K)SRLQT
	220-245-p231 + 235 + 238	T231 + S235 + S238	TREPKKVAVVR[T]PPK[S]PS[S]A(K)SRLQT
	220-245-p231 + 237 + 238	T231 + S237 + S238	TREPKKVAVVR[T]PPKSP[S][S]A(K)SRLQT
386-406-p1^c^	379-411-p396	S396	RENAKAKTDHGAEIVYK[S]PVVSGDTSP(R)HLSNV
	379-411-p404	S404	RENAKAKTDHGAEIVYKSPVVSGDT[S]P(R)HLSNV
386-406-p2	379-411-p396 + 404	S396 + S404	RENAKAKTDHGAEIVYK[S]PVVSGDT[S]P(R)HLSNV
386-406-p3	Same as for 396-406-p2		
407-438-p1^d^	[*U*-^15^N]-labelled non-phosphorylated 407–438		…HLSNVSSTGSIDMVDSPQLATLADEVSASLAK…[*U*-^15^N]
407-438-p2^d^			
407-438-p3^d^			

^a^ Numbering according to tau 2N4R

^b^ [X] amino acid with phosphate group, (X) isotope labelled amino acid; [^13^C_6_,^15^N_2_-Lys] or [^13^C_6_,^15^N_4_-Arg]; black letters are expected peptide after trypsination (based on experiments) and red letters are extended sequence parts

^c^ Labelled standards had the same retention time and since quantification was performed with the intact peptide no distinction was made for different phosphate locations, just the number of phosphate groups

^d^ For these peptides no specific standards were obtained so in these cases surrogate standards were used

^e^ Depending on phosphate location different tryptic peptides were produced, cf. 210-224-p1

#### Evaluation of labelled protein and peptide standards

Since degradation of the protein standards would affect the results, the standards were evaluated by sodium dodecyl sulfate–polyacrylamide gel electrophoresis (SDS-PAGE; Suppl. Figure 3) and LC-MS. No evidence of degradation was observed. Moreover, no trace of unlabelled tryptic peptides could be observed. Because the proteins were digested, the full isoform information cannot be retained, so the link between 0N/1N/2N and 3R/4R was lost. The estimated uncertainty of the equality for the [*U*-^15^N]-protein standards mix was < 20% (see Suppl. Information 3). All phospho-peptide standards (Table 2 and Suppl. Table 2) were also evaluated. For the tryptic peptides included in the comprehensive analysis, no trace of unlabelled peptides was observed. Quality control (QC) samples were used to monitor and calculate the quantitative performance of each peptide (see below).

#### Linearity evaluation

To evaluate the linearity of the method, pooled AD samples were used (since AD had the largest abundance variation). While keeping the amount of all labelled standards constant, the amount sample was varied between 0.125 (1/8) and 4 times the total protein concentration used for the cohorts for the TBS fraction and between 0.0625 (1/16) and 2 times for the SI fraction. Linearity analyses were done in triplicates for the TBS fraction and in duplicates for the SI fraction. Fitting was performed on individual data using the Weighted Linear Regression Excel add-in tool by Real Statistics (www.real-statistics.com), where the weight was 1/x. Throughout the investigated range linearity was good; adjusted r^2^ > 0.91 and in most cases > 0.98 (Suppl. Figure 4 and Suppl. Table 3).

### Analysis overview

**Fig. 2:**
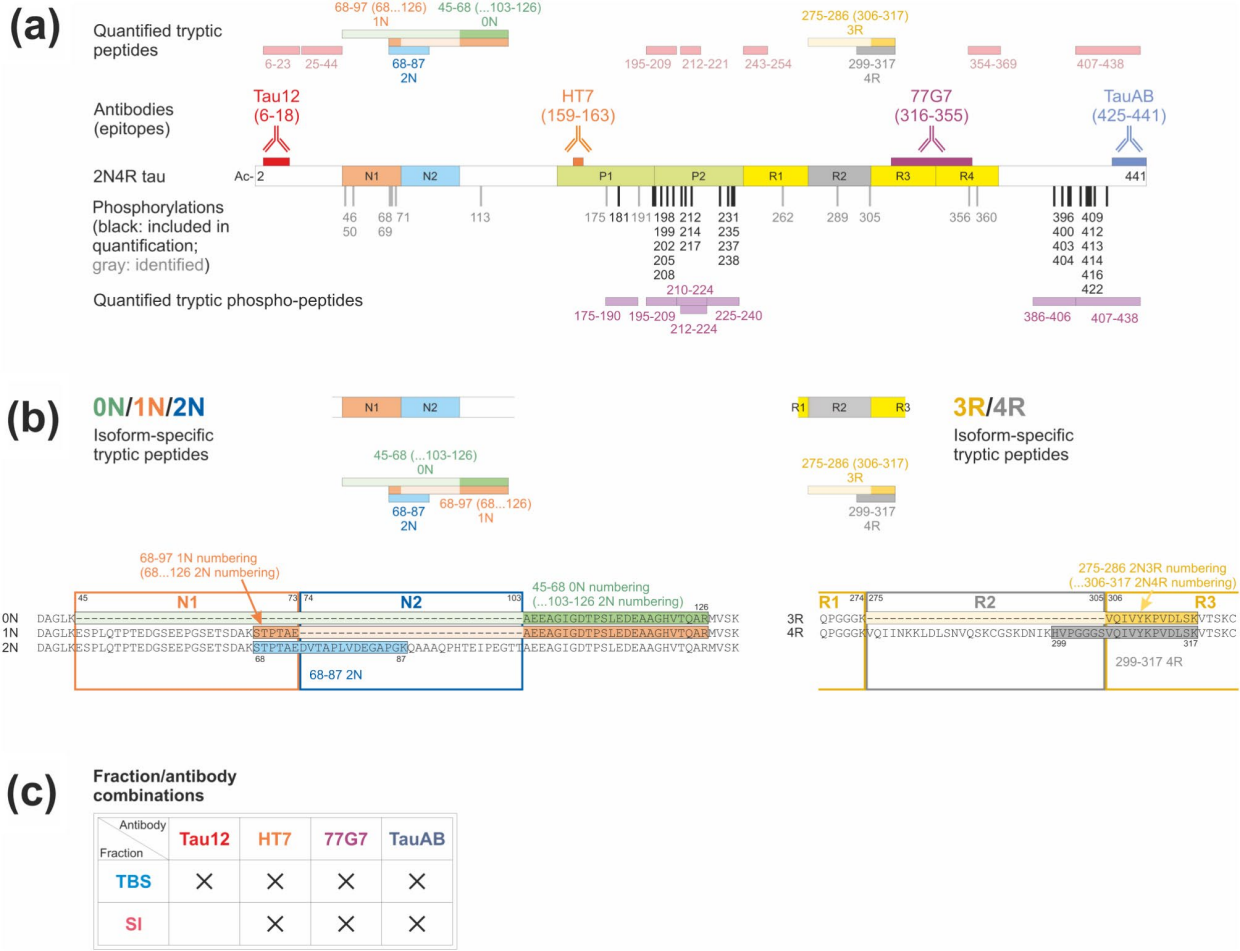
Overview of the neuronal tau protein and the analytical features. (**a**) Schematic of tau protein 2N4R isoform. Indicated are the N1 and N2 inserts, the proline-rich region (P1 & P2), the MTBR (R1-R4), the epitopes of the four antibodies used, the detected phospho-sites, and both the tryptic non-phospho and phospho peptides quantified. (**b**) Details of the isoform specific regions highlighting the quantified isoform specific tryptic peptides. (**c**) The fraction/antibody combinations analysed

Cohort 1 was analysed at seven different occasions, one for each fraction-antibody combination (Fig. 2c). At each occasion, the samples were analysed in a two-step random order to evenly spread the patient groups (see Suppl. Information 4). On all occasions, a set of six QC samples (TBS pool immunoprecipitated with Tau12, see below) underwent the same procedure as the study samples and were used to assess analytical performance as well as to harmonise the data between the acquisitions. QCs, together with blank injections, were spread out evenly over the whole analysis to monitor potential time-dependent drifts and carry-over, respectively. Each data set consisted of 66 injections in a quadrupole-orbitrap MS instrument; 51 samples, 6 QCs, and 9 blank injections. Total acquisition time per set was roughly 100 h.

Cohort 2 was analysed independently using only the HT7 antibody (see below). Here a set of 11 QC samples (same TBS pool as for Cohort 1, immunoprecipitated with HT7) were utilised. Samples were analysed under the same conditions as Cohort 1, except that both the TBS and SI fractions were analysed in turn at the same occasion. This data set consisted of 83 injections, 2 × 30 samples, 11 QCs, and 12 blank injections with a total acquisition time of about 125 h.

#### Tau immunoprecipitation

IP of tau protein in both TBS-soluble and SI brain fractions was performed by coating 50 µL of M-280 Dynabeads (sheep anti-mouse IgG or sheep anti-rabbit from Invitrogen, depending on the host species) with 4 µg of anti-tau antibodies, following manufacturer’s recommendations. In TBS-soluble extracts, a battery of four anti-tau antibodies were selected for IP, each of them having the epitope in one of four different tau regions: Tau12 (N-terminal, amino acids 6–18 in 2N4R Tau, BioLegend), HT7 (mid-region, 159–163, Thermo Fisher Scientific), 77G7 (MTBR, 316–355, BioLegend) and TauAB (C-terminal, 425–441, kindly provided by MedImmune). The epitopes given are those stated by the respective provider. TBS fractions were pooled to create QC samples, which were immunoprecipitated with Tau12 antibody and used all along the analysis (see below). Contrary to TBS-soluble fractions, SI extracts volume (and total protein content) obtained from every 100 mg wet tissue was rather low (approximately 50 µL at 1 mg/mL of total protein concentration from every 100 mg of brain tissue). Due to this limitation, we immunoprecipitated SI fractions with all antibodies but Tau12. Tau12 was excluded based on two main reasons: (1) Tau12 and HT7 provided similar results in TBS-soluble fractions, and (2) tau tangles can get cleavages at both ends of the MTBR, so that using HT7, 77G7, and TauAB would cover the range of peptides within and directly outside the MTBR. Fifty µL of antibody-coated beads were used to IP each sample from TBS-soluble and SI extracts, containing 10 µg or 4.5 µg total protein, respectively. Ten microlitres of the [*U*-^15^N]-protein standards mix (containing 400 fmol protein) were also added to each sample. All samples were brought to 1 mL final volume with phosphate-buffered saline (PBS; 0.01 M phosphate buffer, 0.14 M NaCl; at pH 7.4), including Triton X-100 to a final concentration of 0.05%. Samples were then incubated overnight at 4 °C in a tube roller shaker. Subsequently, an automated magnetic particle processor (KingFisher, Thermo Fisher Scientific) was used to wash and elute the samples. Elution was accomplished using 100 µL of 0.5% formic acid. Finally, sample eluates were dried in a vacuum centrifuge at room temperature and stored at -80 °C.

Negative control experiments were conducted where a non-specific IgG-antibody replaced the anti-tau antibodies. The only cases where any detectable amounts of tau were observed was for peptides from the MTBR in the SI fraction of AD and CBD, where the highest signals were about 10% and 3%, respectively, compared with the levels detected using anti-tau antibodies (data not shown). This was considered to be an acceptable level of unspecific binding.

#### Proteolytic digestion

Proteolytic digestion was performed using trypsin (V5111, Promega). To optimise the digestion efficiency, different amounts of the enzyme were previously tested in a series of identical samples and 100 ng/sample were considered the best amount to be utilise. As previously mentioned, 100 fmol of labelled phospho-peptides were also added to each sample. Samples were then brought to a final total volume of 50 µL with 50 mM ammonium bicarbonate, to allow for a good resuspension and overnight trypsination at 37 °C on a VorTemp 56. The day after, the trypsination was stopped by the addition of 2 µL 10% formic acid. Samples were dried in a vacuum centrifuge and stored at -80 °C pending MS analysis. The use of protein standards and extended phospho-peptides standards improved the quantitative analysis considerably. When initially using tryptic standards, the CVs frequently were 30% or more (data not shown). With the current approach both the standards and the endogenous tau are affected by the variation in digestion efficiency, thus enabling adequate quantitative data.

#### Liquid chromatography-mass spectrometry analysis

NanoLC coupled to electrospray ionisation (ESI) high-resolution hybrid quadrupole–orbitrap MS was performed with a Dionex 3000 system and a Q Exactive (both Thermo Fisher Scientific) in a similar way as previously published [59]. Immunoprecipitated and trypsinated samples were reconstituted in 7 µL 8% formic acid/8% acetonitrile in water. Six µL sample solution was loaded onto an Acclaim PepMap C18 trap column (length 20 mm, internal diameter 75 μm, particle size 3 μm, pore size 100 Å, Thermo Fisher Scientific) for desalting and sample clean-up. Sample loading buffer was 0.05% trifluoroacetic acid in water. Separation was performed by reversed-phase Acclaim PepMap C18 analytical columns (length 150 mm, internal diameter 75 μm, particle size 2 μm, pore size 100 Å, Thermo Fisher Scientific). Separation was performed at a flow rate of 300 µL/min by applying a 50 min long linear gradient from 3 to 40% B. Buffer A was 0.1% formic acid in water and buffer B was 0.1% formic acid/84% acetonitrile in water. The mass spectrometer was set to acquire in the *m/z* range 350–1400 in data dependent mode using higher-energy collision-induced dissociation (HCD) for ion fragmentation. Acquisition settings were the same for MS and MS/MS (mass selection/mass separation) acquisitions for better quality MS/MS data: resolution setting 70 000, one microscan, target values 10^6^, trap injection time 250 ms. An inclusion list for all peptides desired for quantitative analysis was included (see Suppl. Table 2 for peptides included for the final quantification), but when idle the instrument was set to isolate and fragment other ions as well. Dynamic exclusion time was set to only 5 s to maximise chances for identification.

#### Processing of LC-MS and LC-MS/MS data

LC-MS/MS acquisitions of tryptic digests were charge and isotope deconvoluted before submitting searches using PEAKS Studio Xpro (Bioinformatics Solutions) as well as Mascot Daemon v2.6.1/Mascot Distiller v2.6.3/Mascot database search software v2.6.1 (Matrix Science). Searches were made against both UniProt and a custom-made tau-only database. Processing and search settings are described in [59] and Suppl. Information 5.

Quantitative analysis was performed using Skyline software v20.1.0.31 (MacCoss Lab) using the first two isotopes of the precursor ions. Data were then exported to Excel (Microsoft), either as light-to-heavy peptide ratios or as peak areas, for subsequent processing (see Suppl. Information 6 for details).

Depending on the particular peptide, different quantification approaches were used. For the non-phosphorylated tryptic peptides individual tryptic peptides originating from the [*U*-^15^N]-protein standards were used. For most phosphorylated tryptic peptides, peptides originating from the extended phospho-peptides were used. A few phosphorylated peptides did not have the corresponding extended standard so other extended phospho-peptides or peptides from the [*U*-^15^N]-protein standards were used as surrogate standards. The mean of several non-phospho peptides from the [*U*-^15^N]-protein standards was also used as surrogate standard to profile the phospho-peptides along the protein sequence (see Suppl. Information 6). Moreover, several identical aliquots of QC samples (TBS pool) were initially created, then prepared and analysed in sets of six at every different occasion together with the different fraction-antibody combinations. These QCs were then used to normalize all ratios after the final acquisition. See Suppl. Information 7 for details.

### Statistical analysis

Statistical evaluation was performed using Prism v9.4.1 (GraphPad) and JMP v16.0.0 (SAS Institute). Prism was utilized to generate box/scatter plots and correlation data. Data were analysed using Brown-Forsythe and Welch analysis of variance (ANOVA) followed by unpaired t-test with Welch correction for the pairwise comparisons within each fraction-antibody combination. The correlations are Pearson’s r. JMP was employed to generate the correlation scatter plots.

## Results

An overview of the tau protein and a summary of the analytical features investigated is shown in Fig. 2. Cohort 1 was subjected to a thorough investigation involving both soluble (TBS) and insoluble (SI) fractions as well as four different antibodies, while cohort 2 was used to verify previous results and the two fractions were therefore investigated with only the HT7 antibody, chosen as representative of the main findings obtained with all antibodies. Results comparison of Cohorts 1 and 2 are presented at the end of this section.

### Disease-dependent tau isoforms profiles

**Fig. 3:**
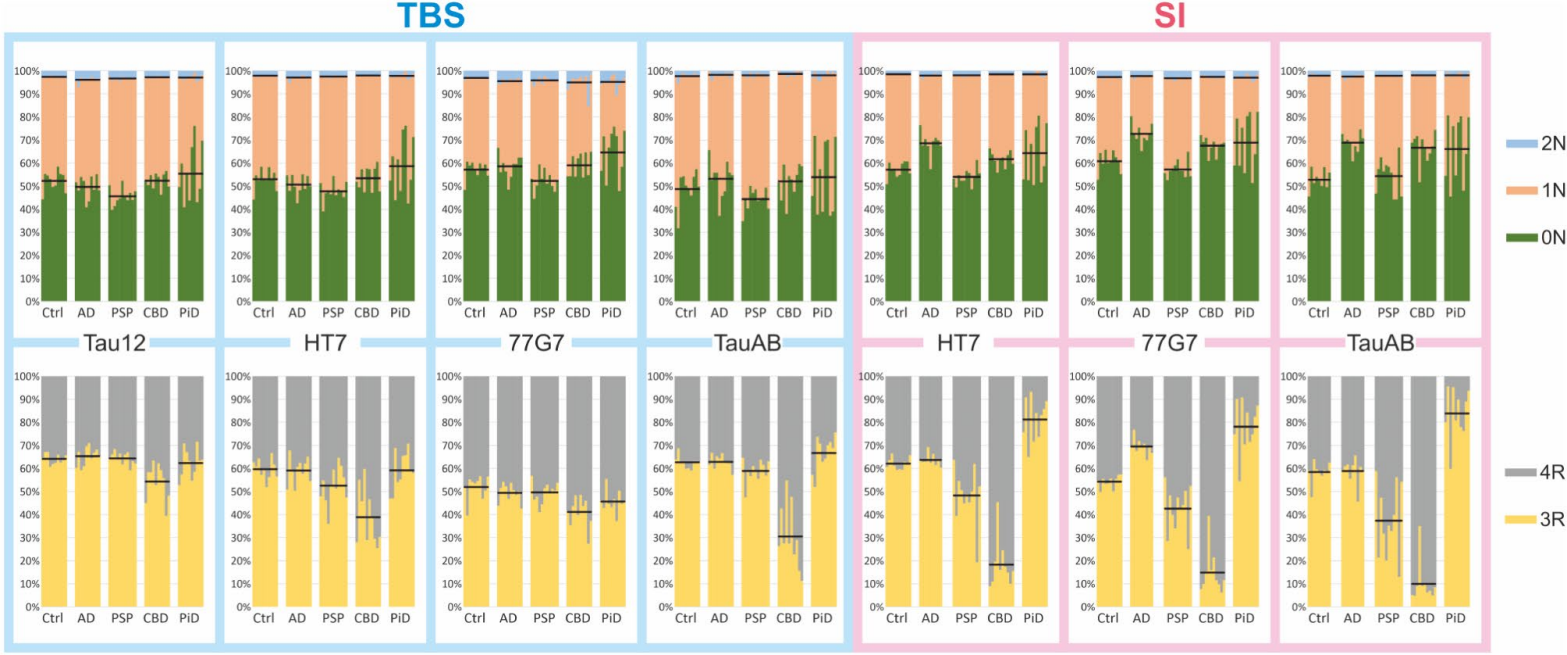
Relative portion of the 0N/1N/2N and 3R/4R isoforms. Each panel represents a fraction-antibody combination as indicated. The individual bars are colour coded for the respective isoforms and grouped according to disease. The black horizontal lines indicate the mean percentage for each isoform. See also Suppl. Table 4 for respective mean values

Frontal grey matter brain samples were analysed using seven different fraction-antibody combinations (Fig. 2c). In general, the isoform measurements, both in TBS and SI fractions, indicate a higher abundance of 0N and 1N species compared with 2N. When examining the 3R and 4R isoforms, the relative abundance of these species showed similar presence of 3R and 4R in control and AD cases, prevalence of 3R in PiD and 4R in CBD and PSP, especially in the SI fraction. As tau protein was digested with trypsin, the link between the N and R isoforms was lost, but within each group they could be assessed and their relative amounts are presented in Fig. 3 and Suppl. Table 4 for all disease and fraction-antibody combinations and differences between the fractions are shown in Suppl. Figure 4.

#### Controls

*N isoforms* In TBS, 0 N isoform was slightly more abundant, especially when immunoprecipitated with 77G7, closely followed by 1 N. The 2 N only isoform represented a small percentage of the total tau amount (0 N/1 N/2 N: Tau12 = 52/45/3%, HT7 = 53/45/2%, 77G7 = 57/40/3%, TauAB = 51/47/2%). The same was observed in the SI fraction, with a moderate increase in the levels of 0 N in relation to 1 N and 2 N (0 N/1 N/2 N: HT7 = 57/41/2%, 77G7 = 61/36/3%, TauAB = 53/45/2%).

*R isoforms* 3R isoform was slightly more abundant than 4R in both TBS and SI. In TBS, IP with Tau12, HT7 and TauAB in approximately a 6/4 ratio in 3R/4R isoforms (3R/4R: Tau12 = 64/36%, HT7 = 59/41%, TauAB: = 62/38%), in contrast with IP with 77G7, which resulted in a nearly 1/1 ratio (3R/4R: 77G7 = 52/48%). In SI, the isoform ratio was virtually the same as in TBS fraction, with a slightly higher abundance of 3R compared with 4R isoform, with 77G7 being closest to a 3R/4R ratio of 1/1 (3R/4R: HT7 = 62/38%, 77G7 = 55/45%, TauAB: = 60/40%).

#### Alzheimer’s disease

*N isoforms* In AD TBS fraction, regardless of the antibody used, the abundance of 0N isoform was higher than 1N. This was especially noticeable when using 77G7 antibody for the IP, similar to what was observed in controls. With values of approximately 5%, 2N was the least abundant isoform in TBS (0N/1N/2N: Tau12 = 50/46/4%, HT7 = 51/46/3%, 77G7 = 59/37/4%, TauAB = 53/45/2%). Interestingly, there was a clear, significant increase of 0N vs. 1N and 2N with all antibodies in the SI fraction when compared with TBS (0 N/1 N/2 N: HT7 = 69/29/2%, 77G7 = 73/25/2%, TauAB = 69/29/2%), see Suppl. Figure 5.

*R isoforms* In TBS 3R was moderately more abundant than 4R, with mean values of around 60%, except when the IP was performed with 77G7, where a 1/1 3R/4R ratio was observed (3R/4R: Tau12 = 65/35%, HT7 = 59/41%, 77G7 = 50/50%, TauAB: = 63/37%). SI HT7 and TauAB showed similar results for 3R/4R isoforms as for their TBS counterparts, which was not the case for 77G7, which resulted in a clear, significant increase in 3R isoform compared with 4R (3R/4R: HT7 = 63/37%, 77G7 = 69/31%, TauAB: = 56/44%), see Suppl. Figure 5.

#### Progressive supranuclear palsy

*N isoforms* In TBS, IP with Tau12 and TauAB resulted in slightly higher 1N than 0N. IP with HT7 yielded similar amounts of 0N and 1N, whereas IP 77G7 resulted in more 0N than 1N. Regardless of the antibody used for IP, 2N was the least abundant isoform. (0N/1N/2N: Tau12 = 46/51/3%, HT7 = 48/49/3%, 77G7 = 53/43/4%, TauAB = 44/54/2%). In the SI fraction, IP with all three antibodies resulted in significantly higher levels of 0N, see Suppl. Figure 5. 2N was always the least abundant isoform regardless of the antibody used (0N/1N/2N: HT7 = 54/44/2%, 77G7 = 57/40/3%, TauAB = 55/43/2%).

*R isoforms* In the TBS fraction, IP with either Tau12 or TauAB resulted in higher levels of 3R, whereas IP with HT7 and 77G7 rendered equal amounts of 3R and 4R isoforms (3R/4R: Tau12 = 64/36%, HT7 = 52/48%, 77G7 = 50/50%, TauAB = 60/40%). In the SI fraction, HT7 pulled slightly higher amounts of 4R than 3R isoform, whereas IP with 77G7 and, in particularly, TauAB resulted in significantly more 4R tau (3R/4R: HT7 = 47/53%, 77G7 = 43/57%, TauAB = 36/64%), see Suppl. Figure 5.

#### Corticobasal neurodegeneration

*N isoforms* In the CBD TBS fraction, there were slightly higher levels of 0N isoform, especially when immunoprecipitated with 77G7, compared with 1N and 2N (0N/1N/2N: Tau12 = 52/45/3%, HT7 = 53/45/2%, 77G7 = 60/36/4%, TauAB = 52/47/1%). In the SI fraction the abundance of the 0N isoform was significantly higher (0N/1N/2N: HT7 = 61/37/2%, 77G7 = 67/30/3%, TauAB = 67/31/2%), see Suppl. Figure 5.

*R isoforms* When it comes to the R isoforms, IP of the TBS fraction with all antibodies except Tau12 resulted in lower levels of 3R compared with 4R, TauAB being the antibody that yielded the largest difference. (3R/4R: Tau12 = 57/43%, HT7 = 40/60%, 77G7 = 43/57%, TauAB = 31/69%). In SI, the 4R prevalence in CBD becomes very apparent with all three antibodies with a significant decrease of 3R (3R/4R: HT7 = 17/83%, 77G7 = 13/87%, TauAB = 8/92%), see Suppl. Figure 5.

#### Pick’s disease

*N isoforms* Regarding N isoforms, 0N was generally the most abundant in TBS, especially when immunoprecipitated with 77G7, and except for TauAB, followed by 1N and 2N (0N/1N/2N: Tau12 = 51/46/3%, HT7 = 55/43/2%, 77G7 = 65/31/4%, TauAB = 44/53/3%). In the SI fraction, the predominance of the 0N isoform became more evident compared with 1N and 2N (0N/1N/2N: HT7 = 62/36/2%, 77G7 = 68/29/3%, TauAB = 65/33/2%); however, due to the individiual heterogeneity no changes were significant, see Suppl. Figure 5.

*R isoforms* For the R isoforms, the TBS fraction of PiD displayed higher levels of 3R compared with 4R, except when immunoprecipitated with 77G7 (3R/4R: Tau12 = 64/36%, HT7 = 58/42%, 77G7 = 46/54%, TauAB = 68/32%). The 3R predominant nature of PiD was significantly more distinguished in the SI fraction, where 3R was remarkably more abundant than 4R (3R/4R: HT7 = 84/16%, 77G7 = 81/19%, TauAB = 88/12%), see Suppl. Figure 5.

### Disease dependent non-phosphorylated tau profiles in the human brain

**Fig. 4:**
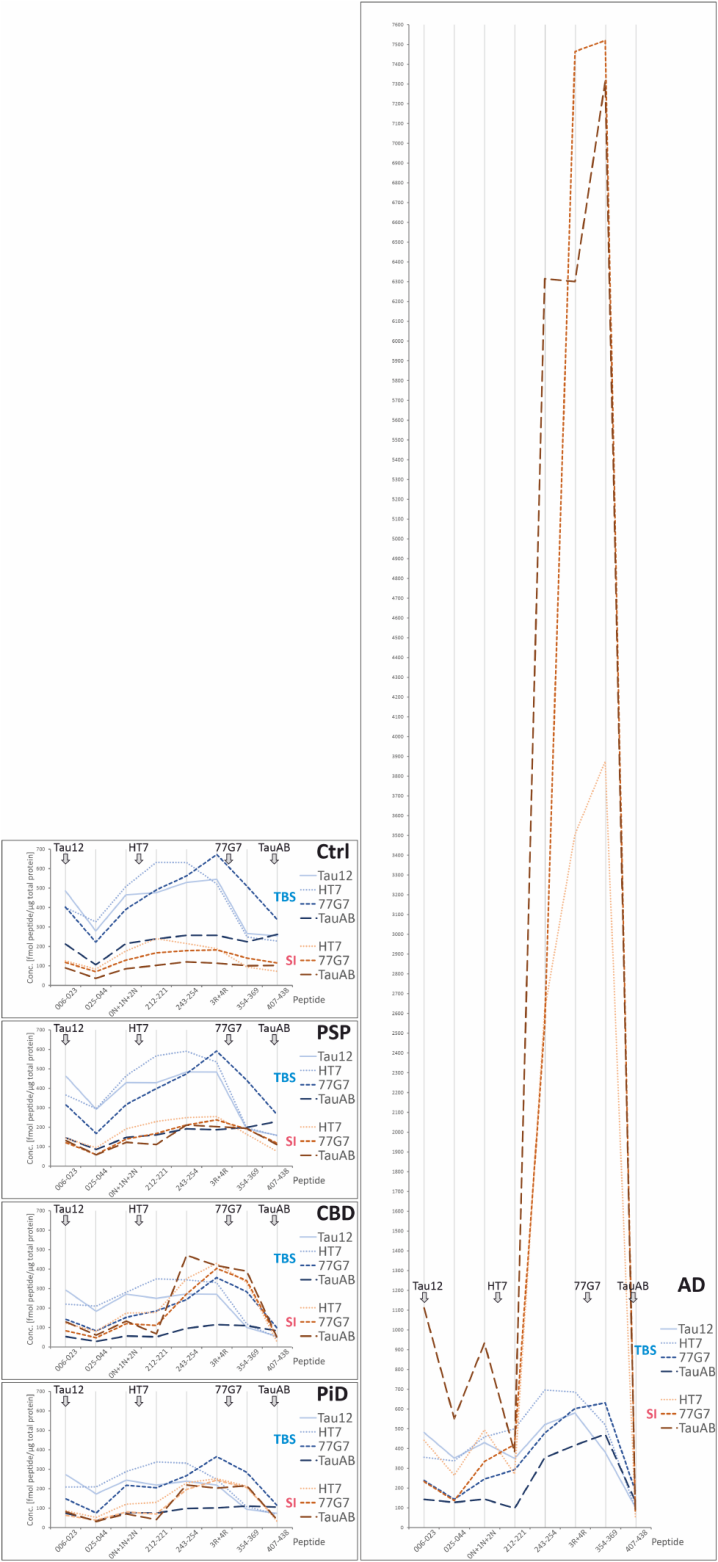
Abundance of monitored tryptic peptides along the tau protein chain. Each panel represents a patient’s group profile as indicated. The profiles are mean values for each fraction-antibody combination. Peptides representing different isoforms (0N/1N/2N and 3R/4R) are summed in the main graphs to avoid dents and their mean shares are shown in the respective side panels. Antibody epitope locations are indicated with grey arrows

Average profiles are displayed in Fig. 4 showing one disease group per panel (an alternative view with one fraction-antibody combination per panel is shown in Suppl. Figure 6, and scatterplots for each quantified peptide are shown in Suppl. Figure 7). These profiles differed depending on the antibody used for IP and the overall effect is easiest to observe in Suppl. Figure 6. To avoid artificial dents in the curves, the signals of the isoform-specific peptides were summed so that the data point 0N + 1N + 2N is the sum of peptides 45–68 0N, 68–97 1N, and 68–87 2N, and 3R + 4R is the sum of 275–286 3R and 299–317 4R peptides. With the exception of AD, tau profiles in TBS showed a clear dependence on the antibody epitope and the peptide abundance generally decreased with distance to the epitope. For AD, the tau profiles for all four antibodies showed an increased presence of MTBR peptides. Of note, the 25–44 peptide was lower than its two adjacent peptides in most profiles; the reason for this is not clear but it may be caused by partial oxidation of methionine, which accounts for a substantial portion of both the brain-derived and the [*U*-^15^N]-protein standard peptide; however, the 6–23 peptide also contains a methionine that also is partially oxidised, making the statement less convincing. This effect has been observed previously [60, 61]. In the SI fraction, tau profiles in tauopathies resulted in high levels of MTBR peptides regardless of the antibody epitope, being especially increased in AD, followed by CBD and PiD, and finally by PSP, which displayed the most level profiles. In control cases, tau profiles in the SI fraction looked comparatively flat (i.e., similar abundance of different tau peptides across the whole tau sequence), except for HT7 and 77G7 which exhibited slightly higher abundance of mid-region and MTBR peptides, respectively.

#### Controls

*TBS-soluble fraction*

After IP with the N-terminal Tau12 antibody, the tau profile between 6 and 23 and 3R + 4R was relatively, flat after which it dropped for peptides 354–369 and 407–438, which had similar levels. IP with HT7 (targeting mid-domain tau) and 77G7 (MTBR) resulted in higher abundance of mid-region and MTBR peptides respectively. On the other hand, while the tau peptide profile between 3R + 4R and 407–438 with HT7 was similar to that of Tau12 (i.e., a steep decrease between 3R + 4R and 354–369 where it levelled out until 407–438), this was not the case for 77G7, which decreased more gradually towards the C-terminus. Finally, IP with TauAB yielded the only (almost) flat profile, mostly unchanged throughout the whole tau sequence. Altogether, the analysed TBS tau pools in control cases seemed to be comprised of a mix of full-length tau and fragments containing N-terminal, mid-region and MTBR regions.

*Sarkosyl-insoluble fraction*

In control SI fractions, tau protein was less abundant than in TBS. Additionally, tau profiles in SI were flatter than their TBS counterparts. IP with HT7, a very subtle increased abundance in mid-region and MTBR peptides was noticeable, especially for the 212–221 peptide. Similarly, with 77G7, most abundant peptides were circumscribed to the mid-region and the MTBR sequence, but this time with the highest abundance shifted toward the MTBR region, where 3R + 4R was slightly higher that the surrounding peptides. Finally, IP with TauAB resulted in a virtually flat tau profile. In summary, the analysed control SI fractions were predominantly comprised of full-length tau and some mid-region and MTBR fragments.

#### Alzheimer’s disease

*TBS-soluble fraction*

In the AD TBS fractions, all tau profiles showed elevated abundance of MTBR peptides, regardless of which antibody was used for the IP. When immunoprecipitated with Tau12, the tau profile was characterized by a lower amount of the 212–221 peptide. This was followed by a peak at 243–254 and 3R + 4R peptides, from which the abundance gradually dropped towards the C-terminus, with 407–438 being the least abundant of all. Tau profiles for HT7 and 77G7 were relatively similar. Both displayed a progressive increase toward the MTBR region. While HT7 showed the highest abundance at 243–254 and 3R + 4R peptides, the maximum abundance peak for 77G7 was at 3R + 4R and 354–369. With both antibodies, the C-terminal 407–438 peptide was low; with HT7 its levels were similar to those of Tau12, while for 77G7, 407–438 was somewhat more abundant. Lastly, the tau profile with TauAB was also prominent in MTBR peptides, and very low in N-terminal, mid-region, and C-terminal species. Notable was the pronounced drop for 212–221. Overall, the analysed AD TBS fractions seemed to be comprised of full-length tau and substantial amounts of MTBR tau fragments.

*Sarkosyl-insoluble fraction*

In the AD SI fraction, IP with all three antibodies resulted in similar tau profiles, which again were characterized by the remarkably prominent abundance of peptides belonging to the MTBR, which were much more dramatically increased than in their TBS counterparts. All three profiles displayed substantially lower levels of N-terminal, mid-region and C-terminal peptides when compared with those belonging to the MTBR. In the mid-region, lower abundance of the 212–221 peptide was seen with HT7 and TauAB, while for 77G7 the more N-terminal peptides showed even lower abundance than the 212–221 peptide. Regardless of epitope, all anti-tau antibody profiles displayed extremely high levels of MTBR peptides, where 3R + 4R and 354–369 were more abundant than 243–254 (for TauAB all MTBR species had similar abundance). In all three profiles, the C-terminal peptide 407–438 was the least abundant tau species, even when immunoprecipitated with TauAB. Thus, AD SI profile was clearly dominated by large amounts of MTBR fragments, also including, to a lesser degree, N-terminal fragments and full-length tau. Overall tau levels in SI fraction from AD were much higher than any other disease group investigated; the abundance of MTBR peptides in AD was 12–72 times higher than in controls and for non-isoform specific MTBR peptides 7–18 times more than in CBD (Suppl. Figure 7 h-k).

In the AD group (both in TBS and SI fractions), 212–221 was typically lower than the adjacent peptides (Fig. 4 and Suppl. Figure 6). It is likely that the observed reduction is due to phosphorylation (see Sect. Phosphorylations below). As aforementioned, the most prominent finding involved the MTBR peptides located between amino acids 243 and 369. Although the effect was visible in the TBS fraction, it was striking in the SI fraction where these peptides were increased more than 10 times even when using antibodies with epitopes outside the MTBR. This effect has also been observed previously [61] and is likely caused by aggregates comprised of several tau peptides belonging exclusively to the MTBR and attached to longer peptides that can be captured by the antibody. Noteworthy is that CBD also exhibited this behaviour, albeit to a lesser degree than for AD (see below).

#### Progressive supranuclear palsy

*TBS-soluble fraction*

Among the tauopathies examined here, PSP is the one with a tau peptide brain profile resembling that in the control group the most (Fig. 4 and Suppl. Figure 6). In TBS, tau profiles with Tau12 and HT7 were very similar. Both were rather flat in the N-terminal and mid-region. This was followed by a very subtle increase for MTBR peptides 243–254 and 3R + 4R when immunoprecipitated with Tau12, whereas with HT7 mid-region and MTBR peptides showed similar abundance. With both antibodies, abundances dropped steeply at peptide 354–369, and continued with a slight decrease towards the C-terminal peptide 407–438 (the least abundant). IP with 77G7 was somewhat similar to those of Tau12 and HT7 but displayed a more pronounced presence of 3R + 4R, which clearly stood out as the most abundant peptides in the profile. From here, the 77G7 profile decreased towards the C-terminal end, but in a more gradual way compared with Tau12 and HT7. Finally, when immunoprecipitated with TauAB, the profile appeared to be for the most part flat, with a subtle increasing trajectory toward the C-terminal end (407–438 was the most abundant), which clearly contrasted with the other antibodies. Altogether, TBS tau in the PSP group seems to be a mixture of full-length tau and fragments containing N-terminal, mid-region, and MTBR regions.

*Sarkosyl-insoluble fraction*

Similar to the TBS fraction, tau peptide profiles in the SI fraction for PSP resemble those seen in the control group. All three antibodies yielded reasonably flat profiles. For all antibodies there was a moderate increase from the N-terminus towards the MTBR region followed by a decrease towards the C-terminus. The differences between the profiles are that HT7 displayed similar amounts of mid-region and MTBR peptides, while TauAB profile seemed to show higher abundance of MTBR and a small drop for 212–221, whereas IP with 77G7 resulted in an in between profile of HT7 and TauAB. Altogether, PSP SI fraction seems to mainly comprise full-length tau and some mid-region and MTBR fragments.

#### Corticobasal neurodegeneration and pick’s disease

*TBS-soluble fraction*

The tau abundance profiles of CBD and PiD in TBS fraction were virtually identical to one another. Additionally, the profiles of CBD and PiD were overall similar to controls and PSP, although the latter two groups having higher levels of all peptides (Suppl. Figure 6). CBD and PiD TBS fractions were comprised of a mix of full-length tau and fragments containing N-terminal, mid-region and MTBR. When using Tau12, CBD and PiD profiles were rather flat (i.e., had similar abundance) across N-terminus, mid-region, and deep into the MTBR (until 3R + 4R), after which they started to gradually drop, with C-terminal 407–438 being the least abundant species. When immunoprecipitated with HT7, mid-region and MTBR peptides were the most dominant, and a similar gradual decrease towards the C-terminal region was observed. With 77G7, MTBR peptides dominated the profile, closely followed by mid-region and neighbouring N-terminal peptides (0N + 1N + 2N). Least abundant peptides were C-terminal and most N-terminal (6–23 and 25–44). Finally, with TauAB, the profile remained virtually flat, showing more or less the same abundance throughout whole sequence, which may indicate that tau species containing the C-terminal region are rather intact and mostly comprised by full-length tau. Overall, CBD and PiD TBS fraction seemed to include a mixture of full-length tau and fragments comprising N-terminus, mid-region, and C-terminus.

*Sarkosyl-insoluble fraction*

Like in TBS, the CBD and PiD tau peptide profiles in the SI fraction displayed the same shape, but this time their peptide abundance was markedly different, with CBD having higher levels of tau. Interestingly, the CBD and PiD profiles showed a clear predominance of MTBR-containing tau species, which resemble those of AD, but comparatively less abundant. Regardless of the antibody used, the N-terminal, mid-region, and C-terminal peptides were the least abundant, whereas peptides belonging to the MTBR, i.e., 243–254, 3R + 4R, and 354–369, were the most abundant with fold changes up to 2 and 4 compared with controls for CBD and PiD, respectively (Suppl. Figure 7 h, k). There was also a small drop for the 212–221 peptide when immunoprecipitated with TauAB and 77G7. Overall, these results suggest that CBD and PiD SI fractions are comprised by a large amount of MTBR peptides and to a lesser degree, full-length tau.

### Phosphorylations

Using the explorative mode of analysis, 36 sites could be identified as harbouring phosphate groups (Fig. 2a) and 16 phospho-peptides were quantified (Table 2). Suppl. Table 5 contains a list of all phospho-peptides identified when using PEAKS Studio Xpro as search engine.

Sometimes it was difficult to pinpoint the exact location of the phosphorylation, since the discriminatory information could rely on as little as two possible fragment peaks. Since peptides with the same number of phosphate groups, but at different positions, also tended to elute at (almost) the same time the fragment spectra could contain peaks from both variants. Nonetheless, with manual evaluation it was possible to confirm the identity of many of the singly, doubly, and triply phosphorylated peptides.

Quantification of the phosphorylated peptides was performed in two ways. Since no useful phosphorylated protein standard was available, to obtain the best relative quantification for comparison between the different patient groups synthetic labelled extended phospho-peptides were used as internal standards. However, since the quantitative method was optimised mainly using TBS fraction material, some phosphorylated peptides later found in SI fraction were not observed and investigated until after spectra from all samples were acquired. Here, the strength of the approach became evident; as spectra were acquired in a data dependent mode, database searches could be performed. Several phospho-epitopes were then detected that were not observed in the method development stages. A number of phospho-peptides were then added to the quantification analysis. For the quantification of these newly found peptides different surrogate internal standards were used (Table 2).

#### Singly phosphorylated peptides

In the TBS fraction, two of the six measured singly phosphorylated peptides were generally lower in CBD and PiD when compared to all other groups; i.e., peptide 175-190-p1 (p181, Fig. 5, Suppl. Figure 8b) and peptide 225-240-p1 (p231, Fig. 5, Suppl. Figure 8i). For HT7 there was a tendency for higher levels in the AD group for all peptides; this could also be seen for TauAB for 210-224-p1 and 225-240-p1. Peptide 212-224-p1 (mostly p217 but also p214, Fig. 5, Suppl. Figure 8 h), was the only mid-region singly phosphorylated peptide being substantially more abundant (4–20 times) in the AD group already in the TBS fraction and regardless of the antibody used for IP.

**Fig. 5:**
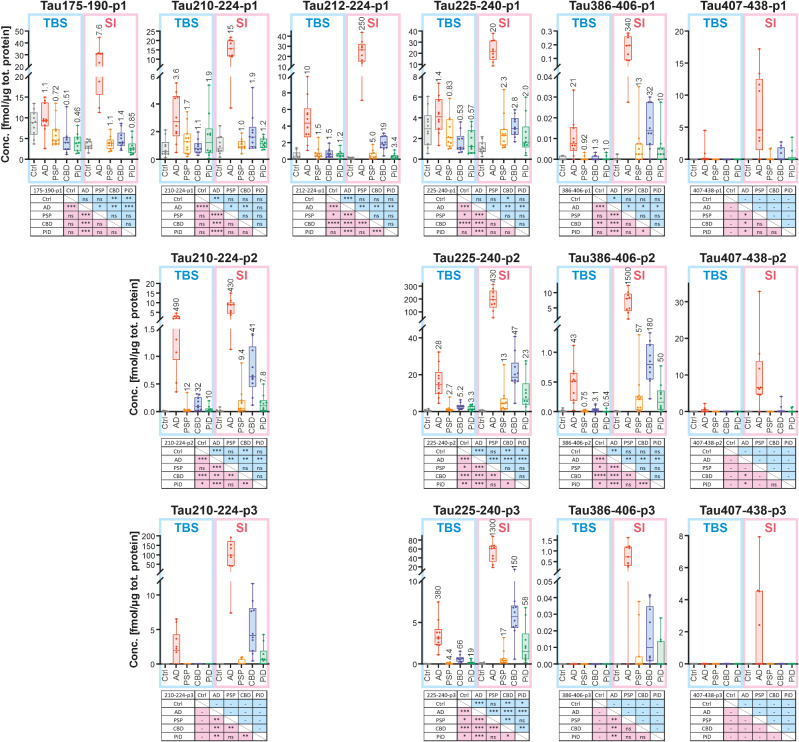
Selected scatter-plots of quantified tryptic phospho-peptides after IP with HT7. Shown are abundancies of peptides carrying one (p1, upper pannels), two (p2, middle panels), or three (p3, bottom panels) phosphorylations. Data is grouped according to fractions. TBS fractions are indicated in blue and SI fractions in pink. Panel top: box/scatter plots with medians, inter-quartile intervals, and min/max intervals indicated. Fold changes of the mean values compared with the controls in respective fraction are indicated for each disease group. Panel bottom: significances according to one-way ANOVA not assuming homoscedasticity (Brown-Forsythe/Welch) followed by unpaired t-test with Welch’s correction. Calculations are only performed within each fraction, upper right (blue) for TBS and lower left (pink) for SI; ns not significant (*p* ≥ 0.05), * *p* < 0.05, ** *p* < 0.01, *** *p* < 0.001, **** *p* < 0.0001, – test not possible to perform due to zero variance in one of the groups compared. See Suppl. Figure 7 for all antibody-fraction combinations analysed

The C-terminal phospho-peptide 386-406-p1 (mainly p396 or p403/404, Fig. 5, Suppl. Figure 8 l) was also more abundant (21 times) in the AD group already in the TBS-soluble fraction compared with the other groups, which did not differ between themselves. For the most C-terminal phospho-peptide 407-438-p1 (p413, p416, or p422, Fig. 5, Suppl. Figure 8o) AD appeared to have higher levels than other groups but the general abundance of these species in TBS was low and the quantitative data less reliable.

In the SI fraction, all singly phosphorylated peptides were much more abundant in AD than in any of the other groups, showing fold changes of more than 1000 compared with the control group, i.e., considerably more than in the TBS fraction (Fig. 5, Suppl. Figure 8b, c, e, h, i, l, o). CBD exhibited the second highest abundance of all groups for all singly phosphorylated peptides (up to 880 times more than for controls) except 195-209-p1 (mainly p202 or p199; Fig. 5, Suppl. Figure 8b, c, e, h, i, l, o). Singly phosphorylated peptides in PSP, PiD, and controls were often comparatively similar with some variation depending on peptide and antibody.

#### Doubly and triply phosphorylated peptides

Several peptides with two and three phosphate groups were detected and quantified (Fig. 5, Suppl. Table 5, Suppl. Figure 8). In the TBS fraction, all peptides carrying two phospho-sites were found increased in the AD group when compared with the other groups (Fig. 5, Suppl. Figure 8d, f, j, m, p, ), with the peptides 210-224-p2 (p212 + p217; 30–500 times higher than controls and 12–70 times more than CBD, Fig. 5, Suppl. Figure 8f) and 225-240-p2 (p231 + p235; 18–66 times higher than controls and 5 times more than CBD, Fig. 5, Suppl. Figure 8j) being the most abundant (Suppl. Figure 9). Also triply phosphorylated peptides observed were markedly increased in AD compared with the other groups in the TBS fraction. Their profiles followed that of doubly phosphorylated, but generally triply phosphorylated peptides appeared to be less abundant than their doubly phosphorylated counterparts, especially in the TBS-soluble fraction where, for example, the C-terminal peptides 386-406-p3 and 407-438-p3 had a relatively low signal also for the AD group (Suppl. Figure 9). The only exception was 210-224-p3, which was similar in abundance to the corresponding doubly phosphorylated peptide (Suppl. Figure 9), but displayed higher fold changes (110–380 times more than controls and 5–7 times more than for CBD; Fig. 5, Suppl. Figure 8j, k). It is noteworthy that also the other tauopathies, in particular CBD, had increased multiple-phospho-peptide levels in TBS compared with controls, displaying moderate to high fold changes (Fig. 5, Suppl. Figure 8).

In the SI fraction, the multiple phosphorylated peptides were even more distinctly elevated and showed higher fold changes in AD than in the TBS fraction (up to more than 1000 compared with controls; Fig. 5, Suppl. Figure 8f, g, j, k, m, n, p, q and 9), but also the other tauopathies had clearly higher levels than controls (Fig. 5, Suppl. Figure 8f, g, j, k, m, n, p, q). As previously observed for singly phosphorylated peptides in the SI fraction, CBD exhibited the second highest abundance after AD for all doubly and triply phosphorylated peptides, followed by PSP and PiD, which generally displayed comparable levels (Fig. 5, Suppl. Figure 8f, g, j, k, m, n, p, q and 9).

#### Particular phosphorylated peptides

Since the tryptic digestion was sometimes incomplete, several sequence stretches were covered by peptides of different lengths. One example is the group 210–221, 210–224, 212–221, and 212–224, which is the result of missed cleavages in the sequence ^210^*SR***T**P**S**LP**T**PPTR*EPK*^224^, where boldface indicates the possible phospho-sites 212, 214, and 217, and a shift between italic and plain text indicate a possible tryptic cleavage site. The phospho-peptide that gave the highest signal was 210–224; this peptide was observed with one, two, or three phosphate groups. The 212–224 peptide was mainly observed with one phosphate group, although the same peptide carrying two phosphate groups was detected as well. When inspecting the MS/MS spectra, the most prominent phospho-site on 212–224 was the one at position Thr-217, but single phosphorylation was also observed at position Ser-214 as well, but not at Thr-212. In contrast, the longer 210–224 form was mainly phosphorylated at position Thr-217, but single phosphorylation was observed to a large extent also at Thr-212. The reason for the Thr-212-phosphorylation being observed only for 210–224 is most likely because the phosphate group at Thr-212 hinders the trypsin cleavage at that position to generate the shorter peptide.

The 195–209 peptide was not visible in TBS because of technical reasons (the peptide is relatively hydrophilic and was borderline to bind to the column), but the MS/MS data obtained from the SI fraction showed that single phosphorylations were most frequent at Ser-202 and Ser-199, followed by Thr-205. Further, Fig. 5 and Suppl. Figure 8d shows that, in the SI fraction, the peptide carrying two phosphate groups was highly increased in AD only, as compared with the same peptide phosphorylated only at one position which seemed to be ubiquitously present in other groups (Fig. 5, Suppl. Figure 8c; data from HT7 and 77G7 only).

#### Non-AD tauopathies

In TBS, all three non-AD tauopathies showed increased levels of multiply phosphorylated peptides compared with controls, especially for CBD. This was not the case for singly phosphorylated peptides. In SI, all non-AD tauopathies generally showed increased phospho-peptide levels compared with controls (Fig. 5, Suppl. Figure 8). This was again particularly evident for CBD, which in SI fraction showed high levels of peptides carrying two or three phospho-groups.

#### Distribution profiles of phosphate groups

Naturally, it was also of interest to compare the relative abundance of the different phosphorylated peptides at different positions. Most phospho-peptides could be quantified, thus relative comparisons across disease groups were made. Similar to non-phospho peptides, the relative tryptic phospho-peptide abundance along the tau protein chain is presented in Suppl. Figure 9. In the TBS fraction, the phospho-tau profile of control, PSP, CBD, and PiD were similar in both shape and peptide abundance, with 175-190-p1 (p181) and 225-240-p1 (p231) being the most prominent peptides. In addition, IP with TauAB in CBD resulted in 225-240-p2 (p231 + p235) being also noticeable and with similar abundance as 175-190-p1 (p181) and 225-240-p1 (p231). On the other hand, AD was characterized by higher levels of doubly and triply phosphorylated peptides compared with all other groups. The most abundant peptides in AD TBS fraction included 175-190-p1 (p181) and 225-240-p2 (p231 + p235), followed by 210-224-p2 (mainly p212 + p217), see Suppl. Figure 9.

In the SI fraction, the controls exhibited a similar profile to that in the TBS fraction but with lower abundance, similarly as previously observed in non-phosphorylated peptide profiles. PSP, CBD, and PiD displayed similar profiles, all characterized by the increased presence of multiply phosphorylated peptides compared with their TBS counterpart and also compared with control SI. The main difference between the groups was the overall peptide abundance, with PSP showing the lowest relative number of phospho-peptides followed by PiD and CBD. Interestingly, as was observed for non-phosphorylated tau profiles, the PSP profile was again similar to that of the control group. The higher abundance of phospho-peptides in CBD was visible in almost all investigated tau species, and generally characterized by the dominance of multiply phosphorylated peptides over their singly phosphorylated counterparts. Phospho-peptides were remarkably more abundant in AD SI than in any other disease group and were also higher than in the AD TBS fraction. The AD SI profile was characterized by the very pronounced presence of 225-240-p2 (p231 + p235) and 225-240-p3 (p231 + p235 + p237/238). They were followed by 175-190-p1 (p181), 210-224-p2 (p212 + p217), 210-224-p3 (p212 + p214 + p217), 386-406-p2 (p396 + p400/403/404), and 386-406-p3 (p396 + p400 + p403/404), see Suppl. Figure 9.

#### Antibody comparison

In TBS, there were clear differences in the non-phosphorylated and phosphorylated tau peptide profiles when using different antibodies for IP, suggesting that although a large part of the soluble tau species likely are full-length tau, a substantial proportion of soluble tau exists also as fragments (Figs. 4 and 5, Suppl. Figures 6–9). The singly phosphorylated peptides from the proline-rich mid-region were generally more abundant when immunoprecipitated with Tau12 or HT7. This contrasted with 386-406-p1 (mainly p396 and p403/404) which was most abundant and with 77G7 followed by TauAB. The most C-terminal, singly phosphorylated peptide, 407-438-p1 (mainly p413, p416, or p422), displayed slightly different results (Fig. 5, Suppl. Figure 8). When using Tau12 or TauAB, 407-438-p1 was observed in the control, AD, and PSP groups and when using 77G7 also in PiD. However, when using HT7 it was detected in only two AD cases (Fig. 5, Suppl. Figure 8).

The results from IP with the C-terminally directed TauAB antibody are noteworthy. In the SI fraction, the relative amounts of phosphorylated mid-region peptides were much higher than when using the mid-region directed HT7 antibody (which has epitope close to most quantified mid-region phospho-peptides) or the MTBR directed 77G7 antibody (Suppl. Figure 8). This effect did not occur in controls and was rather small in PSP, but in PiD, CBD, and AD it became increasingly clear. Even in the TBS fraction, there were noticeably high levels for multiply phosphorylated peptides in AD and CBD.

#### Other detected but not quantified phosphorylations

There were several phosphorylated sites that were detected and identified but not investigated in a quantitative manner (Suppl. Table 5). In the N-terminal region, phosphorylation at Ser-46, Thr-50, as well as at both residues, were detected for the 25–67 peptide (1 missed cleavage) belonging to both the 1N and 2N isoforms. The 1N specific 45–97 peptide (1 missed cleavage) was also found phosphorylated at Ser-68/Thr-69 or both, and Ser-113 was found phosphorylated at peptides from all three N isoforms. In the mid-region, 171–180 phosphorylated at Thr-175 was detected with TauAB in the SI fraction (most likely its absence at the other occasions were because of issues with the binding to the column).

There were also several phospho-epitopes detected in the MTBR that were not quantified. Ser-262/Thr-263 at peptide 258–274 was frequently detected but was excluded from the quantification due to high CV; the same was the case for Ser-305 at the 4R specific 299–317 peptide as well as Ser-324 at the 322–350 peptide. Finally, Ser-289 at the 4R specific peptide 281–290 and Ser-356 at the 354–369 peptide were also detected and identified in a few samples.

### Correlations

Analysis of the correlations between the different tau peptides revealed disease-specific patterns.

#### Correlations of non-phospho peptides

Correlations between non-phospho peptides are shown in Suppl. Figure 10. Most peptides in the control group correlated positively with each other and there was no particular distinction between the TBS and SI fractions. PSP exhibited a similar pattern, although for the SI fraction the more C-terminal peptides in the MTBR (4R and 354–369) had a different behaviour showing lower correlation. In CBD the pattern was similar to that of PSP but C-terminal peptides in the MTBR (4R and 354–369) in SI show weak and moderate negative correlations with TBS fraction peptides. PiD also showed a similar pattern, but here all SI fraction MTBR peptides except 4R behaved differently from the general correlation pattern with a weak negative correlation. Finally, in AD there was a clear separation in correlation between the N-terminal and mid-region peptides compared with the MTBR peptides as well with the C-terminal 407-438-peptide (Suppl. Figure 10b). There was a general pattern of negative correlation between TBS N-terminal and mid-region peptides and SI fraction. N-terminal and mid-region peptides associated positively and strongly within TBS or SI. The same was observed for MTBR species. In the TBS fraction, peptide 407–438 correlated well with the other non-MTBR peptides.

#### Correlations of phospho-peptides

The correlations between phosphorylated peptides are shown in Suppl. Figure 11. For controls, the most common single phosphorylations 175-190-p1 (p181) and 225-240-p1 (p231) correlated best with 225-240-p2 (p231 + p235), *r* = 0.67–0.98 and *r* = 0.63–0.92, respectively. For AD, most peptides correlated well; especially within the SI fraction. For PSP, CBD, and PiD the situation was somewhere between that of controls and AD, with relatively good correlations within the SI fraction, and more variable otherwise. PSP in TBS and SI fractions generally showed positive and moderate correlations within and with each other, whereas in CBD, this only happened within each fraction but not between TBS and SI. Finally, PiD showed a stronger correlation in the SI fraction.

#### Correlations of phospho-peptides to non-phospho peptides

The correlations between phosphorylated and non-phosphorylated peptides are shown in Suppl. Figure 12. In controls, the only noticeable pattern was that the TBS phospho-peptides exhibited higher correlations with all non-phospho peptides than the SI phospho-peptides did. In AD, most phospho-peptides correlated best with the MTBR non-phospho peptides, especially in the SI fraction. Most phospho-peptides in TBS and SI anti-correlated with non-phospho peptides outside the MTBR in TBS (Suppl. Figure 12b). Different tau regions, N-terminal to mid-region, MTBR, a C-terminal region, associated very differently with the phosphorylated peptides.

For PSP, the pattern was more similar to that of controls, but with weaker correlations. In general, TBS phospho-peptides had low or positive correlation with most non-phospho peptides. Most SI phospho-peptides, on the other hand, had low or negative correlation with most non-phospho peptides. The main exception to this was that most SI phospho-peptides (and to a lesser degree TBS phospho-peptides) correlated positively with the SI MTBR non-phospho peptides 299–317 4R and 354–369 while the SI 275–286 3R peptide showed no correlation. CBD showed a similar pattern as PSP but with more pronounced positive correlations, where most SI phospho-peptides correlated strongly with all SI MTBR peptides except 3R, also 243–254. The SI phospho-peptides also had a weak positive correlation with SI N-terminal to mid-region non-phospho peptides. In PiD, most phospho-peptides had, with some exceptions, low or negative correlation with most non-phospho peptides in TBS. TBS 175-190-p1 (p181) and TBS 225-240-p1 (p231) in general correlated positively with non-phospho peptides. The most noticeable feature, however, was the strong positive correlation of SI phospho-peptides with all SI MTBR non-phospho peptides except 4R. This was also the case for several TBS phospho-peptides.

**Fig. 6:**
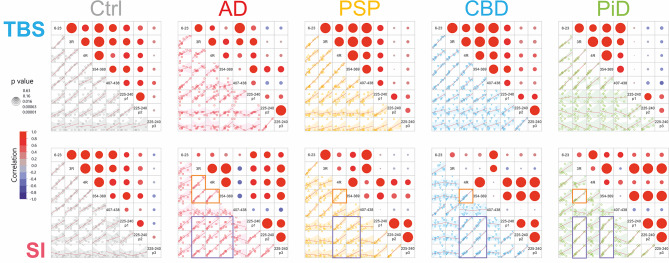
Selected correlations from IP with 77G7 for both the TBS and SI fractions. Correlations are Pearson’s r. Indicated with orange boxes are the high correlation with the respective disease-associated major isoform with a non-specific MTBR peptide for the SI fraction. Purple boxes indicate the high positive correlation with MTBR peptides and phosphorylated peptides in the proline-rich mid-region in the SI fraction. In AD, all MTBR peptides correlate with each other and with the phosphorylated peptides. In PSP and CBD the only the 4R specific and the non-specific peptides correlate, while in PiD the situation is the opposite with only the 3R specific and the non-specific peptides correlating. Correlation coefficient values are indicated by the colour scale and significance by size of the circles

As an example, the correlations between selected phospho-peptides and selected non-phospho peptides for both TBS and SI fractions immunoprecipitated with 77G7 are shown in Fig. 6. Highlighted are the high positive correlations between phosphorylated peptides in SI and the disease associated MTBR peptides in SI, i.e., non-isoform-specific 354–369 for all diseases (AD *r* = 0.72–0.89, *p* < 0.03; PSP *r* = 0.55–0.77, *p* < 0.09; CBD *r* = 0.91–0.97, *p* < 0.001; PiD *r* = 0.95–0.96, *p* < 0.0001), 4R for AD (*r* = 0.75–0.95, *p* < 0.02), PSP (*r* = 0.63–0.87, *p* < 0.04), and CBD (*r* = 0.94–0.98, *p* < 0.001), and 3R for AD (*r* = 0.76–0.90, *p* < 0.02) and PiD (*r* = 0.97–0.99, *p* < 0.0001). No such correlations were observed in TBS.

### Comparisons with the validation cohort

To validate our findings, an independent cohort (Cohort 2) consisting of frontal grey matter from control (*n* = 10), sporadic AD (*n* = 10), and CBD cases (*n* = 10) was analysed using HT7 (results with this antibody are representative of the main findings obtain with all other used antibodies). The results from Cohort 2 were compared with those of Cohort 1 and are presented in Suppl. Figures 13–19.

#### Tau isoform profiles

Tau isoform profiles are shown in Suppl. Figure 13, where the two cohorts are displayed side by side. There were no or small differences with the exception of the 3R/4R ratio in the TBS soluble fraction for CBD, which was clearly lower in Cohort 1 compared with controls and AD, while for Cohort 2 there was no difference.

#### Non-phosphorylated tau

Profiles for non-phosphorylated peptides are shown in Suppl. Figure 14 and scatter plots in Suppl. Figure 15. Although not completely identical for the two cohorts, the TBS soluble profiles for controls, AD, and CBD exhibit the same general pattern where the most prominent feature was found in the increased abundance of the MTBR peptides for the AD group. Similarly, for the SI fraction the profiles are qualitatively very similar for both cohorts. The noticeable differences are that for Cohort 2 the control group is lower, while AD and CBD are higher.

#### Phosphorylated tau

Scatter plots comparing the two cohorts are shown in Suppl. Figure 16, where the two cohorts are displayed side by side and distribution profiles are shown in Suppl. Figure 17. Also here the general pattern is similar for the two cohorts, particularly in the SI fraction, with the main difference being the control and AD profiles in the TBS soluble fraction which were higher in Cohort 1 (Suppl. Figure 17).

#### Correlations

Correlation data for comparison of the two cohorts are presented in Suppl. Figure 18, which displays side by side the same selected correlations as in Fig. 6. For the TBS soluble fraction, the two cohorts exhibited comparable pattern. Also for the SI fraction the bulk of the data was similar; in particular did both the 3R and 4R peptides in AD but only the 4R peptide in CBD correlate with the phospho-peptides. The main discrepancy was that in Cohort 2 all peptides correlated positively in AD, while in Cohort 1 the non-MTBR peptides 6–23 and 407–438 did not correlate with the other peptides displayed. A comparison of all correlations is shown in Suppl. Figure 19; the correlations largely exhibited similar results in both cohorts, although there were also some differences between some groups of peptides. Most notable was the shift from negative to positive correlation for the C-terminal 407–438 peptide with the MTBR and phosphorylated peptides in the SI fraction of AD.

## Discussion

In this study, we report a detailed mass spectrometric characterization of tau protein in brain tissues of different tauopathies using two cohorts. Combining IP with nanoflow liquid chromatography coupled to high resolution mass spectrometry, we developed and validated a multiplex method capable of detecting and quantifying tau protein isoform-specific peptides and phosphorylated and non-phosphorylated tau species in brain extracts. Employing different antibodies targeting tau protein at different regions, we isolated tau protein brain extracts from control, AD, PSP, CBD, and PiD cases, which we subsequently analysed using our mass spectrometry multiplex method. Our findings indicate that,

1. tau protein tryptic peptide profiles differ among tauopathies and control cases, but also between soluble and insoluble fractions, in both scenarios AD profiles being the most outstanding among the studied groups,
2. tau (assessed by the abundance of MTBR-tau peptides) aggregation appears to be especially pronounced in AD, particularly in the SI fraction,
3. although much more pronounced in AD, both aggregation and hyperphosphorylation is present in other tauopathies; in particular CBD,
4. tau hyperphosphorylation in AD is especially prominent for the doubly phosphorylated 225–240 peptide, and
5. the proportion of 3R and 4R tau isoforms clearly distinguishes the tauopathies

### Antibody epitope-dependent tau profiles in the human brain

In the TBS fractions, the peptide abundance was dependent on the antibody epitope and generally decreased with distance to the epitope (Fig. 4 and Suppl. Figure 6). This is consistent with a relatively high amount of tau protein fragments of various lengths accompanying full-length tau. Additionally, and regardless of the antibody used for IP, all disease tau profiles in TBS displayed the same general shape (just differing in terms of peptide abundance). There was however a noticeable exception; when IP was performed with TauAB, tau profiles were rather flat (except for AD) indicating that TBS-soluble tau species containing the C-terminal region are to a high degree full length tau, while for the other antibodies there was a distinct decrease in abundance when moving towards the C-terminus. This can be interpreted as shorter C-terminus containing fragments end up in the CSF and/or are processed to even smaller fragments that evade detection. Only in the AD group, the TBS peptide abundance profiles always showed an increase for MTBR peptides. Since the MTBR peptides were more abundant also when the antibody epitope lied outside this region, this can be interpreted as aggregates comprising tau fragments are captured, where at least one of the aggregated fragments covers the epitope. This is also in line with the results from the Western blots (Suppl. Figure 1).

In the SI fraction, tau peptide profiles across diseases can be organized into three groups based on their overall shape. First, control and PSP profiles were comparatively flat regardless of the antibody used for IP, which is interpreted as their respective SI fraction being comprised to a large extent of full-length tau and some mid-region and MTBR fragments. The second tau peptide profile group comprises CBD and PiD. Their profiles displayed the same overall shape, which regardless of the antibody used was characterized by a noticeable presence of MTBR peptides. For PiD, the increase is not more pronounced than for PSP but more confined to the MTBR. CBD, on the other hand, displayed more overall peptide abundance in SI than PiD and PSP, an increase present only in the MTBR. This indicates that SI fractions of CBD and PiD contain tau aggregates comprised by MTBR fragments and in the case of CBD, a lesser extent of full-length tau. Finally, AD can be considered a third separate group as the levels of MTBR peptides were much higher than the other groups (approximately 18 and 35 times higher than in CBD and PiD, respectively). From our results, this effect was stronger with TauAB than HT7, and similar for TauAB and 77G7. Considering that TauAB is a C-terminal antibody (epitope at amino acids 425–441), it would be expected that the high levels of MTBR peptides in the SI fraction of AD would be accompanied by a corresponding high abundance of C-terminal non-phosphorylated tau peptides. However, non-phosphorylated C-terminal peptides were low in abundance. Moreover, the ability of TauAB to capture non-phosphorylated MTBR tau peptides in AD SI fraction was even more pronounced for phosphorylated peptides. These results cannot be linked to a potential higher affinity of the antibody. In fact, TauAB did not show this effect in the TBS fraction when compared with the other antibodies. The reason(s) why TauAB apparently precipitates more efficiently tau protein in AD SI requires further investigation.

Taken altogether, tau protein aggregates were markedly more abundant in AD than in any other of the studied disease groups. This could be the result of a difference in tau protein across tauopathies to form abnormal aggregates, to hijack other tau molecules in a prion-like manner, and on its ability to propagate from cell to cell. Additionally, sporadic AD is a comparatively slow progressing disease, presenting a total disease duration (including preclinical AD) between 12 and 25 years [62], contrasting with PSP and CBD, which are all characterized by a faster progression [63, 64]. Thus, tau protein in AD might have more time to aggregate and accumulate prior to death, which combined with the increased production rate induced by amyloidosis, may result in the higher tau content observed in AD brain. This hypothesis is also consistent with our results indicating that a very substantial part of all tau protein content in AD was comprised by MTBR-containing aggregates.

### N isoforms

The N isoforms are comparatively less studied than R isoforms. N isoforms have been suggested to be associated with the cellular distribution of tau [65], but not much is known about their actual abundance across different tauopathies, especially in non-AD tauopathies, as most attempts to quantify these isoforms have been performed with controls and AD cases [39, 42]. In AD, the general agreement is that 1N is the most common isoform, closely followed by 0N and finally 2N, which is by far the least abundant [66, 67]. In general, 0N isoforms were slightly more abundant than 1N in the TBS fraction, and this was more pronounced in SI, particularly for AD, CBD, and PiD. Since only frontal grey matter tissue was available, we cannot conclude whether this N isoform pattern is exclusive or not of this brain region. Monitoring the isoform abundance is also affected by truncations and phosphorylations in the region, which have not been assessed. Thus, it would be of great interest to further investigate the levels of N isoforms in more detail, in other brain regions, and in other cohorts to fully characterize their levels across physiological and pathological scenarios.

### R isoforms

Contrary to the N isoforms, R isoforms are more widely studied due to the differential immunoreactivity pattern of tau pathological inclusions to anti-3R and − 4R antibodies across tauopathies [14, 56]. According to these criteria, CBD and PSP are 4R tauopathies, whereas PiD and AD are 3R and 3R/4R tauopathies, respectively. Generally, our data shows that these differences were not overt in the TBS fraction, whereas the SI fraction reflected the 3R/4R immunoreactivity of the histopathological inclusions. Looking closer, for controls and AD our results indicate that these groups may have slightly more 3R than 4R isoforms. These results do not challenge the 3R/4R nature of AD, and may just simply suggest that the 3R/4R ratio in healthy adults and AD cases might not be exactly 1:1, as also previously reported [68]. Furthermore, we expected the R isoforms pattern in PSP to be similar to that of CBD; however, this was not the case. In the TBS fraction, 4R isoforms were slightly less abundant than 3R. Conversely, in the SI fraction, 4R isoforms were more abundant than 3R (significant increase compared with TBS for 77G7 and TauAB antibodies), but the 4R nature of PSP was not as overt as in CBD. These findings are likely related to the different distribution of tau lesions in CBD and PSP [69–71]. This is in line with PSP containing less MTBR aggregates than CBD and looking more like a control, which, together with the high abundance of full-length tau in the extracts, might have confounded the results showing a less pronounced abundance of 4R. Results for PSP and CBD are in line with those reported recently by Horie et al. who found that 4R were increased in CBD and, to a lesser extent, PSP compared with controls [72]. However, they also found 4R increased for one out of two peptides in AD, which we did not. There can be several explanations for this discrepancy. We measure the most C-terminal of the 4R peptides, 299–317, while Horie et al. measures 275–280 and 282–290 [72]. The different parts of tau may be subject to PTMs or truncations modulating the abundance of specific peptides. Results for AD, CBD, and PiD are also in line with the data reported by Zola et al., albeit they obtained more distinct 3R/4R ratios, possibly because of the stronger centrifugation [73]. Moreover, the 3R/4R ratio differed depending on antibody used indicating that the ratio varies depending on the type of fragment. Both SI PSP and CBD showed a trend of more 4R the more C-terminal the antibody epitope was. AD and controls have similar 3R/4R ratios in both TBS and SI for all antibodies except for 77G7, which in SI fractions shifts the ratio of the AD group from 50 to 70% 3R. This can be interpreted as tau species comprising only MTBR in AD aggregates contain 3R to a higher degree than other tau species, aggregated or not. Together, the collected data reveals the complex nature of both physiological and pathological processing that result in this variety of tau species.

### Phosphorylated and non-phosphorylated tau

One of the aims of this study was to investigate if the phosphorylation pattern of tau differs across tauopathies and if some specific phosphorylated residues possess biomarker potential. Based on our results, a few general observations can be made. (1) Phosphorylation levels, like non-phosphorylated tau, were generally more pronounced in SI than in TBS, especially for AD. (2) In the SI fraction, doubly and triply phosphorylated peptides were generally more abundant than their respective mono-phosphorylated counterparts in all studied tauopathies. (3) All observed multiply phosphorylated peptides were distinctly more abundant in AD regardless of antibody used. (4) In the SI fraction, quantified phosphorylated peptides were essentially in all cases significantly increased in AD compared with all other groups. (5) Most of the phosphorylated residues identified were detectable in all tauopathies; even in the control group many were detected. Double and triple phosphorylated tau species were not always detected in control cases but they were frequently measurable across AD and non-AD tauopathies. It was also clear that multiply phosphorylated peptides were more abundant in the SI fraction strongly associated with aggregated tau. Interestingly our findings were rather disparate with those of Zola et al. who measured higher abundance of several multiply phosphorylated peptides in the sarkosyl-soluble fraction than in the SI fraction [73].

One of the main findings of this study is that phosphorylated tau content (as well as non-phosphorylated tau) is notably higher in AD than in controls and non-AD tauopathies. To better understand the results from tau phosphorylation, it is important to take into account the non-phosphorylated tau peptides. If we combine tau measurements in TBS and SI in each group, including phosphorylated and non-phosphorylated tau species, it becomes even more evident that total tau content appears to be substantially more abundant in AD brain than in controls, CBD, PSP, and PiD. As mentioned above when discussing non-phosphorylated tau, this may be due to a difference in tau protein across tauopathies to form aggregates and/or propagate, combined with the fact that sporadic AD has a slower progression and longer disease duration than PSP, CBD, and PiD. We also found that the non-phosphorylated tau protein extracted from AD brain was mostly comprised by MTBR-containing tau fragments which leaves an important question unanswered: where are the “missing” N-terminal, mid-region, and C-terminal fragments? A likely explanation would be a connection between these missing fragments and CSF tau biomarkers. Under physiological and pathological conditions, tau content in CSF is mostly comprised by a pool of soluble N-terminal and mid-region tau fragments, all the way from the N-terminus until approximately amino acid 255 with a marked abundance drop after about amino acid 224 [31, 38, 60]. This coincides with the beginning of the MTBR fragments that dominate AD brain protein extracts. Thus, it is reasonable to speculate that the characteristic increase in CSF p-tau species (i.e., p181, p217, p231, and p235) and total-tau in AD [25, 53, 74, 75] are the result of N-terminal and mid-region truncations [76] on the highly abundant tau pool present in AD brain, and their subsequent migration into CSF. In fact, our results demonstrate that the highly AD specific p-tau residues measured by CSF biomarkers (i.e., p-tau181, p-tau217, p-tau231, and p-tau235) are also highly increased in AD brain compared with controls and non-AD tauopathies, especially in SI fractions. In contrast, biomarker literature consistently reports unchanged CSF tau biomarkers levels in non-AD tauopathies, displaying levels comparable to those of healthy individuals [30, 31, 77]. Our results show that when compared with AD, TBS and SI tau profiles in controls and non-AD tauopathies appear at a first glance relatively similar in terms of shape and content. Thus, this may indicate that tau amount, at least in the studied non-AD tauopathies, might not substantially differ from controls, ultimately rendering the similar levels of CSF t-tau described in the literature [30, 77]. We have also demonstrated that the phosphorylated residues measured by published CSF biomarkers (p-tau181, p-tau217, p-tau231, and p-tau235) are indeed measurable in brain and in many instances, significantly increased in brain extracts from non-AD tauopathies when compared with controls. However, the abundance of this phosphorylated species in non-AD tauopathies is overshadowed, especially in SI fractions, by the very pronounced levels found in AD. These results suggest that the AD-specific increases in CSF p-tau biomarkers might be a by-product of the overall increase in amounts of phosphorylated tau protein in AD rather than actual disease specificity per se of a given amino acid residue, as non-AD pathologies do also display increased levels of p-tau residues in brain, but to a much lesser degree than AD. Thus, we suggest that it is the abundance rather than the disease-specificity that ultimately determines the ability of phosphorylated tau residues to discriminate AD from non-AD tauopathies, both in brain and CSF. This is further supported by immunohistochemistry in brain tissues from AD and non-AD tauopathies, which consistently show the high immunoreactivity of tau inclusions to antibodies such as AT8 (p-tau202/205) or PHF1 (p-tau396/404), ultimately demonstrating that highly phosphorylated tau is a major pathological component of both AD and non-AD tauopathies [12]. In addition, MS studies have reported C-terminal tau peptides in CSF from approximately amino acid 386 to the C-terminus [31, 61, 78]. However, the abundance of C-terminal species in CSF is lower than those containing N-terminal and mid-region. A possible explanation for this is the intense proteolytic processing which characterizes this relatively short tau region, including cleavages at aspartic acid 421 and glutamic acid 391 [79], which combined with the action of trypsin, may cause this region to be hard to detect and quantify in CSF.

Of note, singly phosphorylated species at Thr-212 and Thr-217 were more frequently observed than Ser-214 and thus we can speculate that they are more frequently present, although we cannot say if any of the positions is preferentially phosphorylated first when multiple phosphate groups are present. On the other hand, we observe a sequential phosphorylation on the peptide 225–240 which can carry one, two or three phosphorylations at position Thr-231, Ser-235, and Ser-237Ser-238, respectively (it was not possible to distinguish between the adjacent sites Ser-237 and Ser-238 from the MS/MS data). This has been previously observed and reported by Hanger et al. [41]. For this peptide, we can see how phosphorylation is a sequential event, as phosphorylation at position Ser-235 was never detected unless a priming phosphorylation at Thr-231 was present, and phosphorylation at Ser-237/Ser-238 mainly appeared when the other two were present. However, due to the properties of the digestion and susequently the resulting peptides, it is rather possible that a phosphorylation at Ser-235 combined with none at Thr-231 would produce the tryptic peptide 231–240, which is very hydrophilic and less likely to bind to the column. Therefore, the lack of such a peptide cannot support any conclusions on the order of phosphorylation events. However, the order of phosphorylation events was previously shown in vivo in CSF, where the sequential occupancy of the phospho sites, first at Thr-231 and then at Ser-235 was shown to be associated with disease progression and useful to stage preclinical AD [53]. Although a sequential phosphorylation event was especially evident on the peptide 225–240, other serine and threonine clusters located in other peptides, such as 210–224, 386–406, and 407–438, may undergo a similar process. At any rate, these results suggest that multiple phosphorylation is a prominent feature of AD and pathological tau accumulation. Interestingly, in the TBS fraction, the discriminative power of the peptide increases as phospho-sites accumulate; peptides carrying one phosphorylation do not typically discriminate between groups whereas peptides carrying two or three phosphorylations, clearly separated the AD group from the other diseases as well as controls.

### Correlations

As aforementioned, when compared with AD, TBS and SI tau profiles in controls and non-AD tauopathies are similar in terms of shape and content. For example, in TBS almost all peptides correlate well with each other in all four groups. However, upon closer examination some differences can be discerned. In TBS, control and PSP profiles are similar to each other, whereas CBD and PiD profiles are also quite alike. Also, in SI, controls and PSP are relatively similar; there is a small contribution from aggregated species that is not present in controls and the shorter mid-region peptides (i.e., those immunoprecipitated with HT7) in controls correlate less with the other peptides. In SI, CBD and PiD are again closest to each other with the main difference being the higher amount of aggregated species in CBD. Correlation-wise they are more similar to AD than to PSP and controls with tight correlations within the MTBR, but not between the MTBR and the other regions. CBD and PiD exhibit a few differences in the correlation patter indicating differences in the cleavage which could be an important key to address the pathological processes involving tau protein.

The PSP, CBD, and PiD brains were selected to not include any visible AD co-morbidity. Nevertheless, there were increased levels of phosphorylated tau, particularly doubly and triply phosphorylated peptides, in CBD, PSP, and PiD as compared with controls. The correlation data shown in Fig. 6 clearly connects the respective specific isoforms with phosphorylations in the SI fraction but not in the TBS soluble fraction. In the SI fraction, phospho-peptides correlated well with 4R (but not 3R) for PSP, strongly for CBD and strongly with 3R (but not 4R) in PiD. This points at an obvious connection of phosphorylations and disease-specific aggregates and that no chemical evidence of AD co-morbidity is observed.

### Validation cohort

Comparison of the two cohorts showed the similarity in both qualitative and quantitative terms. The main findings from Cohort 1 could be replicated in Cohort 2, including the very high abundance of MTBR peptides in the SI fraction of AD, obtained with an antibody with epitope outside that region, and the likewise increased abundance in CBD. Moreover, the high correlation between MTBR peptides and phospho-peptides in the SI fraction was verified in AD as well as in CBD; in the latter group the isoform specificity of 4R was also verified since the 3R peptide did not correlate in either cohort.

Although the data from Cohort 2 largely verifies the analysis of Cohort 1, there were some minor discrepancies, for example, the higher abundance of all non-phosphorylated peptides for AD and CBD in the SI fraction of Cohort 2, and the shift in correlation of the most C-terminal peptide 407–438 with MTBR and phosphorylated peptides in the SI fraction for AD in Cohort 2. The brain extraction for the two cohorts was performed at two separate occasions and the ultracentrifuge and related accessories used for the SI fraction was exchanged in between. It is well known that of all steps in the sample handling and analysis, the tissue itself and the protein extraction gives the largest variation [80] and while most subsequent steps can be normalised by using internal standards, this is not feasible for the initial tissue handling. We chose to normalise the obtained tau signal to total protein content, but precise quantification from separate extractions is still difficult to obtain. From the acquired data it appears that the separation into soluble and insoluble tau was more efficient for Cohort 2 than for Cohort 1 In Suppl. Figure 14, it can be seen that the relative amount of tau in the control group was clearly lower in the SI fraction of Cohort 2. Another difference was found in the correlation data (Suppl. Figures 18 and 19), where the C-terminal 407–438 peptide, which is weakly negatively correlated with MTBR and phosphorylated peptides in the SI fraction of AD in Cohort 1, was instead positively correlated in Cohort 2. A reasonable explanation for this is that there is a higher portion of soluble tau in the SI fraction of Cohort 1 than in Cohort 2. This is further supported by the 3R/4R pattern of CBD in TBS fractions from Cohort 1, where 4R isoforms are more abundant than in Cohort 2. This may suggest a less efficient fractionation and the subsequent presence of some CBD SI fraction, very high in 4R tau species. Other correlation data points in this direction as well, hinting at a sharper separation of the two fractions for Cohort 2. While this minor contribution does not affect the main findings of the present study, the impact on the low-abundant C-terminal peptide is important to note.

In summary, comparison of the two independent cohorts clearly shows that the general pattern and main findings are reproducible, as clearly exemplified by Suppl. Figures 14 and 17, but caution should be taken when interpreting the behaviour of low abundant tau species.

### Strengths and limitations

In order to compare tau protein across tauopathies, a brain region pathologically relevant for both AD and non-AD tauopathies had to be selected. Therefore, the brain material used in this study was limited to frontal grey matter and consequently, potential differences in tau pathology in other regions could not be assessed. Thus, it would be of great interest to expand the current findings by investigating tau protein isoforms and phosphorylation in other brain regions. However, due to the different regional spreading pattern of tau pathology across tauopathies, studying other brain regions might impede the fair comparison between tauopathies, as some regions will be spared of tau pathology while others are greatly affected. The separation into TBS and SI fractions was not 100% complete and was sharper in Cohort 2. While this does not alter the main findings, it would be benficiary to improve this step to obtain more clear-cut data and avoid possible misinterpretations. Another limitation can be related to the use of trypsin. While trypsination of immunoprecipitated tau protein is essential in order to quantify full-length tau and high molecular mass tau fragments using MS, this is limited by the fact that the ability for trypsin to cleave after lysine or arginine when they are followed by a phosphorylated residue is significantly affected. Thus, it is feasible to hypothesize that other phosphorylated tryptic species exist. Moreover, the use of trypsination, while necessary for quantifying all tau species regardless of their molecular mass, hamper the identification of potentially relevant endogenous tau cleavages connected to disease-specific proteolytic processing [38, 81]. Due to this methodological limitation, further studies comparing different enzymes are warranted.

Key strengths of the present study include the use of two brain cohorts and the extraction and analysis of two different brain fractions for each case - TBS-soluble (containing soluble tau fragments, full-length and oligomeric tau) and SI (comprising insoluble tau aggregates) - a strategy that allows the investigation of a wide spectrum of tau species in brain. However, while this protocol has been previously shown to effectively separate these two populations of tau species [57] and although differences are clearly present between the two fractions, the 100% separation between the two fractions cannot be assumed. The total brain tissues used, which include a large number of non-AD tauopathy cases (all same region) all well-characterized neuropathologically, represent another strength of this study, together with the use of four different antibodies for IP, which removes any potential target bias induced by the epitopes. Ultimately, the use of various different heavy-labelled standards allowed for a detailed quantification and characterization of tau proteoforms in each tauopathy.

## Conclusions

Taken together, this is the first study to simultaneously quantify tau protein isoforms, non-phosphorylated and phosphorylated tau protein in both TBS and SI fractions from different tauopathies, specifically AD, PSP, CBD, and PiD, and control cases. We show here that the SI fraction has higher abundancy of phosphorylations and MTBR region peptides when compared with the TBS fraction, possibly reflecting an interphase between small soluble oligomers and larger aggregates as those found in immunohistochemical inclusions. Moreover, we demonstrate that abnormal levels of phosphorylation and aggregation occur in non-AD tauopathies as well as in AD, but both are manifold more pronounced in AD, to such a degree that it becomes a distinct feature of AD pathology. Most importantly, no specific phosphorylation appears unique for any of the studied non-AD tauopathies. The studied phosphorylations were detectable in all the disease and control groups, which strongly suggest that it is the abundance of phosphorylated peptides rather than the disease-specificity of a given phosphorylated residue what ultimately determines its biomarker potential. In conclusion, this study shows that tau hyperphosphorylation and aggregation are prominent features of AD and that multiply phosphorylated peptides are highly disease-specific species for AD with superior biomarker potential compared with singly phosphorylated forms.

### Electronic supplementary material

Below is the link to the electronic supplementary material.

**Supplementary Material 1:** Figure 10

**Supplementary Material 2:** Figure 11

**Supplementary Material 3:** Figure 12

**Supplementary Material 4:** Figure 19

**Supplementary Material 5:** Figures

**Supplementary Material 6:** Information

**Supplementary Material 7:** Table 3

**Supplementary Material 8:** Tables

## Data Availability

All data and material (de-identified patient information) will be made accessible by the authors upon reasonable request.

## Abbreviations

Aβ: Amyloid-β
AD: Alzheimer’s disease
ANOVA: Analysis of variance
CAA: Cerebral amyloid angiopathy
CBD: Corticobasal neurodegeneration
CSE: Cerebrospinal fluid
EDTA: Ethylenediaminetetraacetic acid
EGTA: Ethylene glycol-bis(β-aminoethyl ether)-*N,N,N’,N’*-tetraacetic acid
ESI: Electrospray ionisation
FGM: Frontal grey matter
HCD: Higher-energy collision-induced dissociation
LC: Liquid chromatography
MS: Mass spectrometry
MS/MS: Mass selection/mass separation
MTBR: Microtubule-binding region
NFT: Neurofibrillary tangle
NIA-AA: National Institute on Aging and the Alzheimer’s Association
NINCDS: National Institute of Neurological and Communicative Disorders and Stroke
PBS: Phosphate-buffered saline
PiD: Pick’s disease
PSP: Progressive supranuclear palsy
p-tau: Phosphorylated tau
PTM: Post-translational modification
QC: Quality control
QSBB: Queen Square Brain Bank for Neurological Disorders
SDS-PAGE: Sodium dodecyl sulfate-polyacrylamide gel electrophoresis
SI: Sarkosyl-insoluble
TBS: Tris-buffered saline
tris: Tris(hydroxymethyl)aminomethane
t-tau: Total tau
UCL: University College London
