# Supplementary material for: Tau protein profiling in tauopathies: a human brain study
Source: Mol Neurodegener. 2024 Jul 19;19:54. doi: 10.1186/s13024-024-00741-9 (PMC11264707; doi:10.1186/s13024-024-00741-9)
Supplement: Supplementary file 5 — Supplementary Material 5: Figures [file 13024_2024_741_MOESM5_ESM.docx]

**Supplementary Figure 1.** Western blots of TBS fraction (5 µg/lane, a) and SI fraction (1 µg/lane, b) using PHF-1 (directed at tau phosphorylated at Ser396/Ser404). Ctrl; AD, CBD. Bands marked GAPDH (glyceraldehyde-3-phosphate dehydrogenase) below each panel represent signals obtained from reprobing the PHF-1 blots with GAPDH as loading/transfer control.


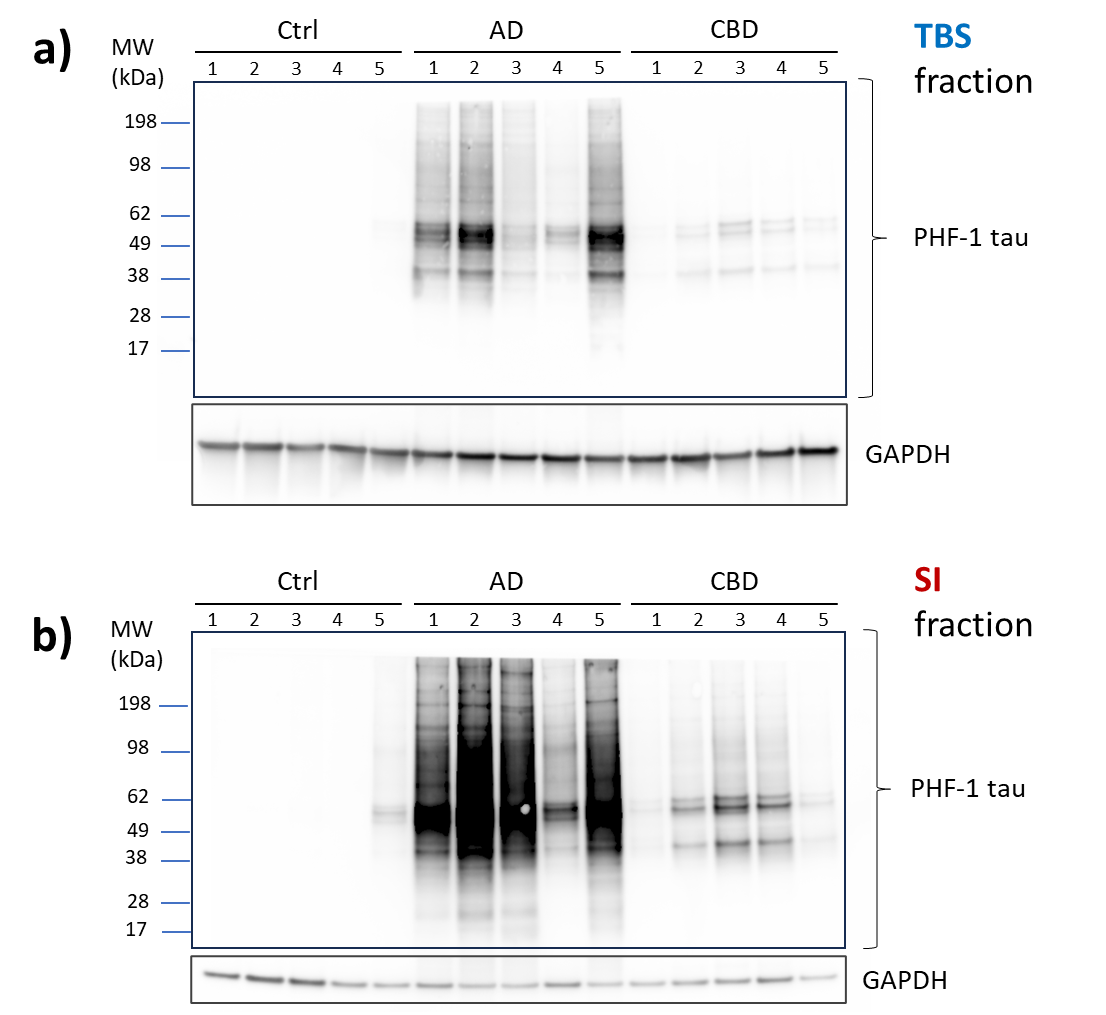


**Supplementary Figure 2**. The six CNS specific splice variants of the tau protein. Tau protein is always observed with the initial methionine scissored off and the N-terminal alanine acetylated (Ac).

**Supplementary Figure 3.** Coomassie stained SDS-page gel of the purified [*U*-^15^N]^-^labelled tau isoforms.

**Supplementary Figure 4.** Linearity test for all peptides with a labelled standard. Different amounts of pooled AD sample was immuprecipitated using HT7, while keeping the amount of isotope-labelled standards constant. y-axes are measured light peptide-to-heavy peptide ratios (L/H) and x-axes are relative amount of brain sample where 1 corresponds to the amount used in the study sets (indicated with an arrow in each panel). Dots are mean of three or two analyses for TBS and SI fraction, respectively (see Suppl. Table 3 for all individually measured ratios), and lines are weighted fits (1/x) using individual data. a) non-phospho peptides in TBS fraction, b) non-phospho peptides in SI fraction, c) phospho peptides in TBS fraction, and d) phospho peptides in SI fraction.

**
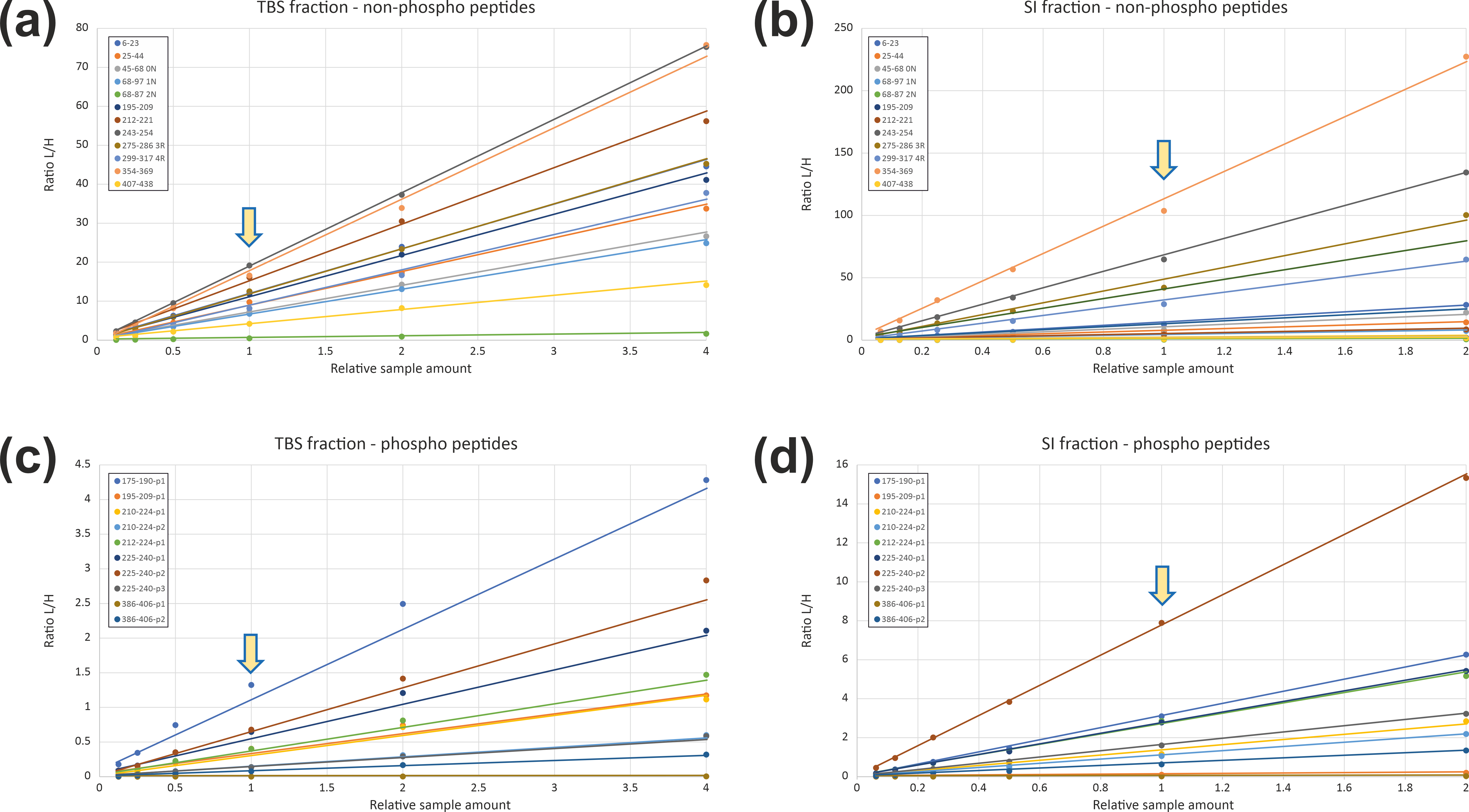
**

**Supplementary Figure 5.** Relative portion of the 0N/1N/2N and 3R/4R isoforms. Each panel compares the TBS and SI fractions for the antibodies HT7, 77G7, and TauAB for either the N- or the R-isoforms. Significant changes for 0N and 3R (which is equal to the change of 4R) between the fractions are indicated: * p<0.05, ** p<0.01, *** p<0.001, **** p<0.0001. The individual bars are colour coded for the respective isoforms and grouped according to disease. The black horizontal lines indicate the mean percentage for each isoform. See also Suppl. Table 3 for respective mean values.


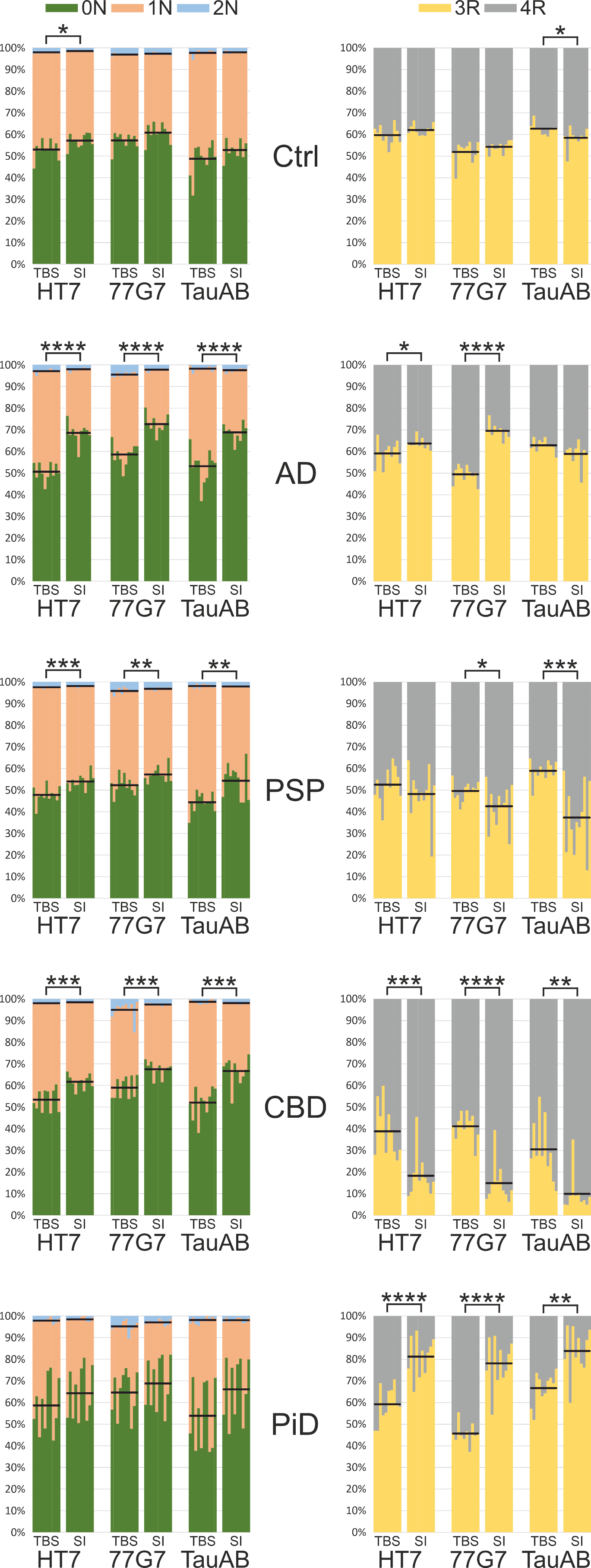


**Supplementary Figure 6.** Abundance of monitored tryptic peptides along the tau protein chain. Each panel represents a fraction-antibody combination as indicated. The profiles are mean values for each patient group. Peptides representing different isoforms (0N/1N/2N and 3R/4R) and are summed; see Fig. 3 for their respective shares. Antibody epitope locations are indicated with grey arrows.


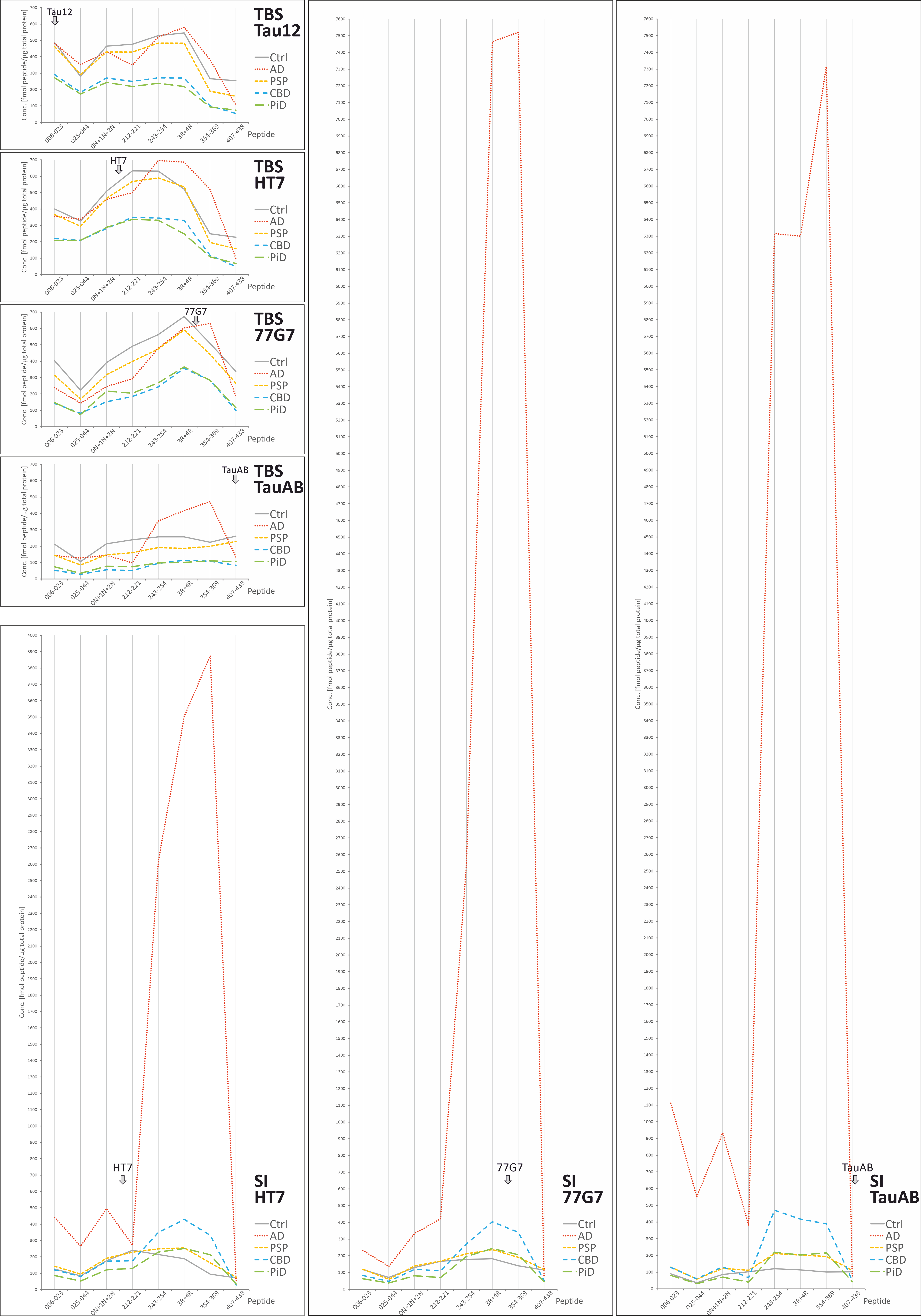


**Supplementary Figure 7.** Scatter-plot of non-phosphorylated peptides and isoforms. Quantified tryptic non-phospho peptides. Data is grouped according to fraction-antibody combination. TBS fractions are indicated blue and SI fractions pink. Top: box/scatter plots with medians, inter-quartile intervals and min/max intervals indicated. Middle: significances according to one-way ANOVA not assuming homoscedasticity (Brown-Forsythe/Welch) followed by unpaired t-test with Welch’s correction. Calculations are only performed within each fraction-antibody combination; ns not significant (p≥0.05), * p<0.05, ** p<0.01, *** p<0.001, **** p<0.0001. Bottom: fold changes of the mean values compared with the controls in each fraction-antibody combination.

**a)**

**b)**

**c)**

**d)**

**e)**

**f)**

**g)**

**h)**

**i)**

**j)**

**k)**

**l)**

**Supplementary Figure 8**. Scatter-plot of quantified tryptic phospho-peptides. Data is grouped according to fraction-antibody combination. TBS fractions are indicated blue and SI fractions pink. Top: box/scatter plots with medians, inter-quartile intervals and min/max intervals indicated. Middle: significances according to one-way ANOVA not assuming homoscedasticity (Brown-Forsythe/Welch) followed by unpaired t-test with Welch’s correction. Calculations are only performed within each fraction-antibody combination; ns not significant (p≥0.05), * p<0.05, ** p<0.01, *** p<0.001, **** p<0.0001. Bottom: fold changes of the mean values compared with the controls in each fraction-antibody combination. For some phospho-peptides the signals were below detection limit for all samples in several groups, making it impossible to perform an adequate statistical evaluation (all these peptides were the ones for which no labelled standard was added).

**a)**

**b)**

**c)**

**d)**

**e)**

**f)**

**g)**

**h)**

**i)**

**j)**

**k)**

**l)**

**m)**

**n)**

**o)**

**p)**

**q)**

**Supplementary Figure 9.** Relative tryptic phospho-peptide abundance along the tau protein chain. The left top panel summarises the positions of the phosphate groups found in the exploratory analyses. Each of the other panels represents a patient group. For the left panels all y-scales have the same interval distance while for the right panel (showing the full AD profiles) the scale is compressed. The profiles are the mean values for each fraction-antibody combination.

**
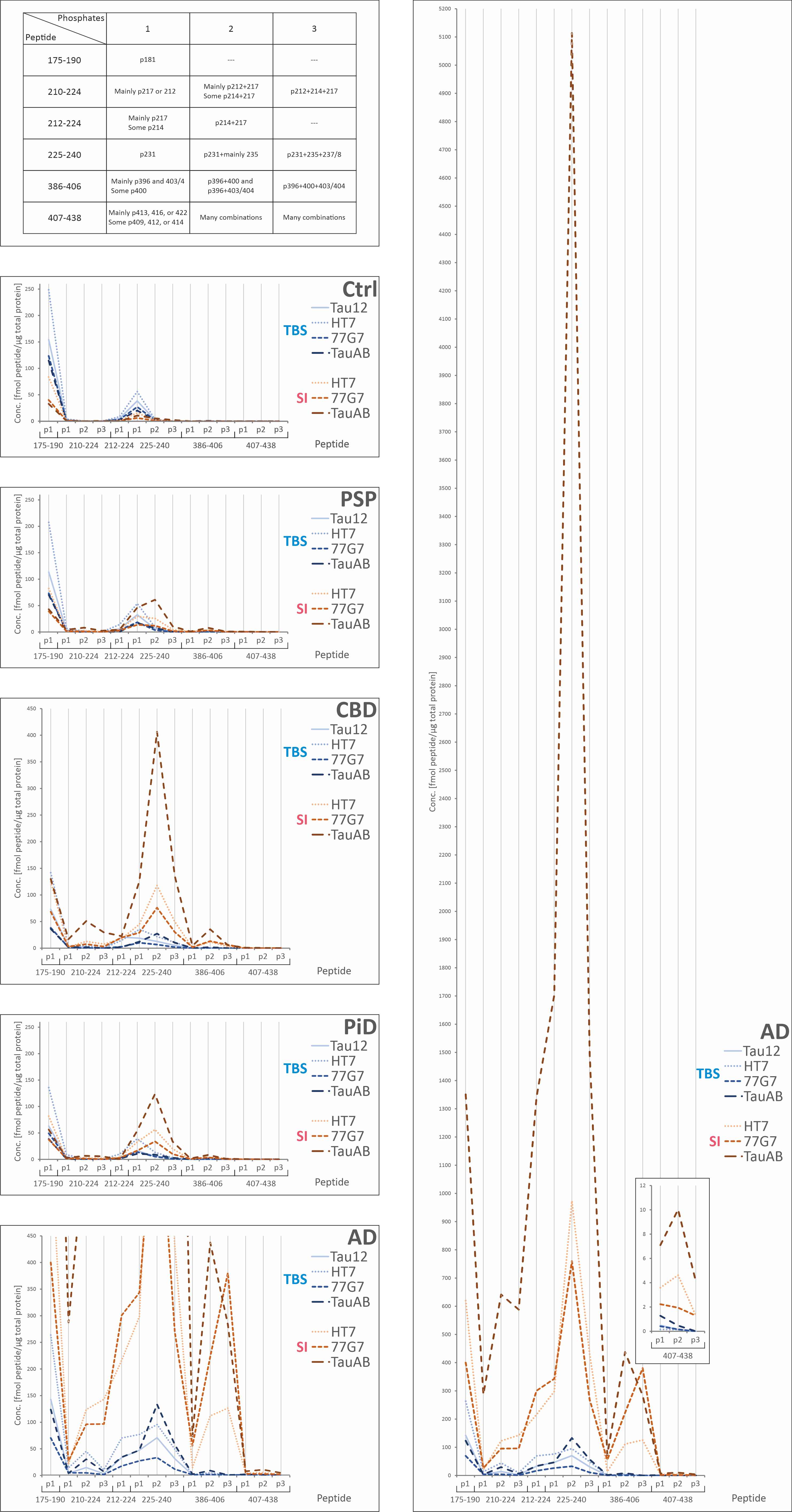
**

**Supplementary Figure 10 (separate Excel-file).** Separate correlations between non-phosphorylated tryptic peptides for each patient group. The data is divided by extraction fraction, peptide (N-terminal to C-terminal), and capture antibody (epitope order). Red indicates +1 positive correlation, white 0 no correlation, and blue -1 negative correlation. (a) controls, (b) AD, (c) PSP, (d) CBD, and (e) PiD.

**Supplementary Figure 11 (separate Excel-file).** Separate correlations between phosphorylated tryptic peptides for each patient group. The data is divided by extraction fraction, peptide (N-terminal to C-terminal), and capture antibody (epitope order). Red indicates +1 positive correlation, white 0 no correlation, and blue -1 negative correlation. (a) controls, (b) AD, (c) PSP, (d) CBD, and (e) PiD.

**Supplementary Figure 12 (separate Excel-file).** Separate correlations between non-phosphorylated and phosphorylated tryptic peptides for each patient group. The data is divided by extraction fraction, peptide (N-terminal to C-terminal), and capture antibody (epitope order). Red indicates +1 positive correlation, white 0 no correlation, and blue -1 negative correlation. (a) controls, (b) AD, (c) PSP, (d) CBD, and (e) PiD.

**Supplementary Figure 13.** Relative portion of the 0N/1N/2N and 3R/4R isoforms. Each panel compares the Cohort 1 (#1) and Cohort 2 (#2) for the N- or R-isoforms and the TBS and the SI fractions. Significant changes for 0N and 3R (which is equal to the change of 4R) between the fractions are indicated: * p<0.05, *** p<0.001. The individual bars are colour coded for the respective isoforms and grouped according to disease. The black horizontal lines indicate the mean percentage for each isoform. See also Suppl. Table 3 for respective mean values.

**
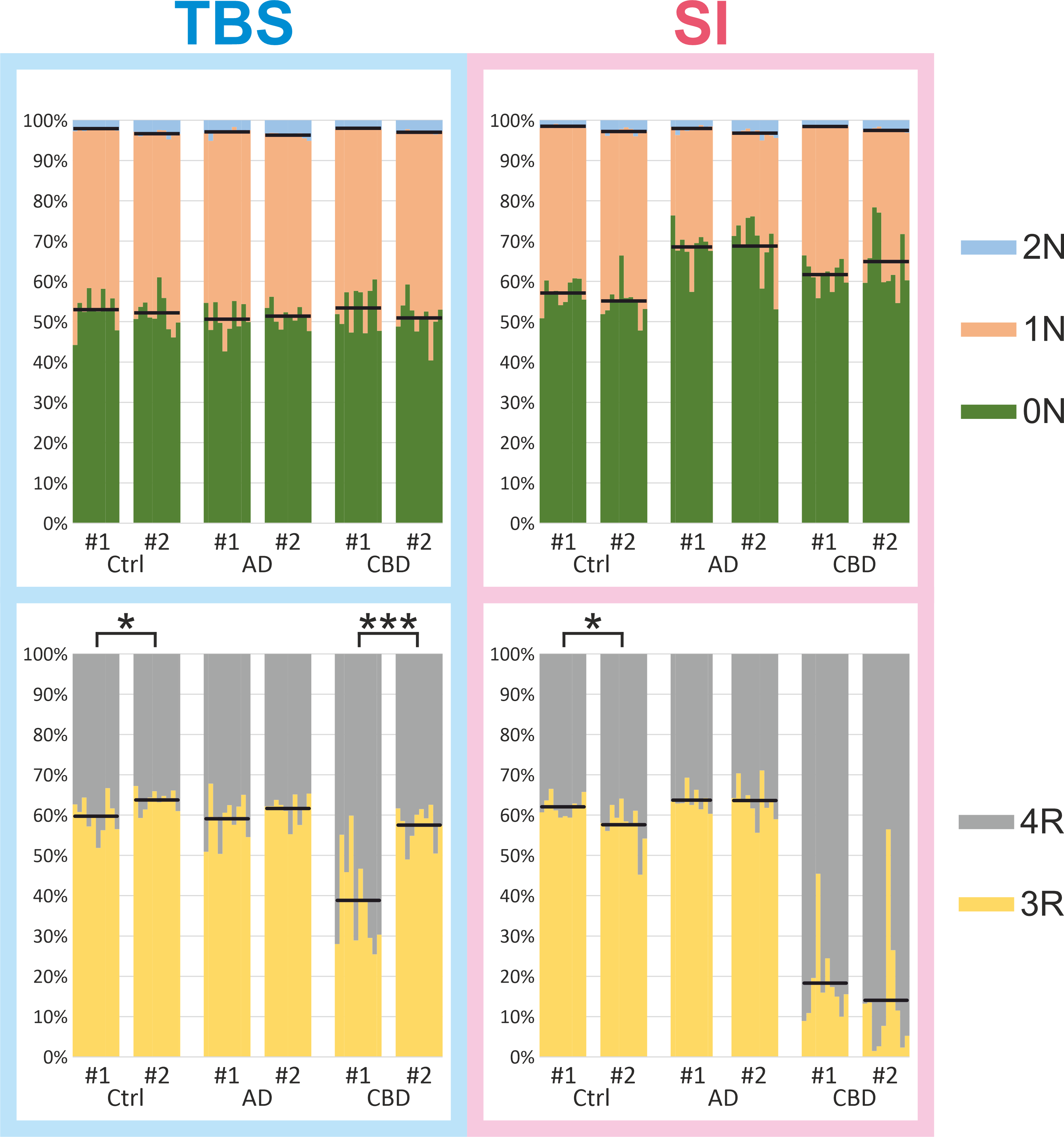
**

**Supplementary Figure 14.** Comparison of the abundance of monitored tryptic peptides along the tau protein chain for Cohort 1 (#1) and Cohort 2 (#2) immunoprecipitated with HT7. The top panels show the TBS fraction for the respective cohorts and the bottom panels the SI fraction. The profiles are mean values for each patient group. Peptides representing different isoforms (0N/1N/2N and 3R/4R) and are summed; see Supplementary Fig. 12 for their respective shares. Antibody epitope locations are indicated with grey arrows.

**
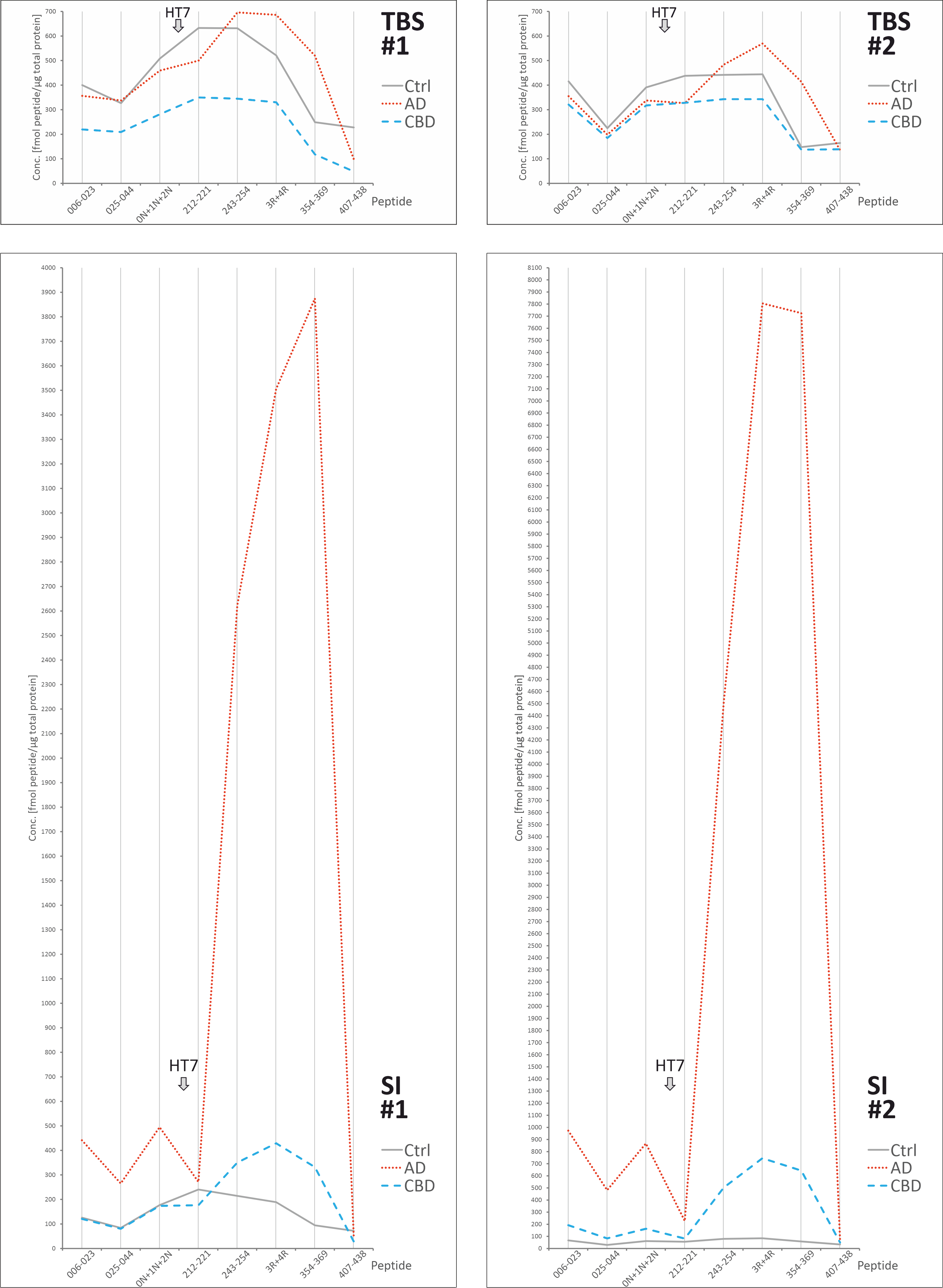
**

**Supplementary Figure 15.** Scatter-plots of non-phosporylated tryptic peptides for both cohorts. Data is grouped according to fraction-cohort combination; antibody used for cohort comparison was HT7. TBS fractions are indicated blue and SI fractions pink. Top: box/scatter plots with medians, inter-quartile intervals and min/max intervals indicated. Middle: significances according to one-way ANOVA not assuming homoscedasticity (Brown-Forsythe/Welch) followed by unpaired t-test with Welch’s correction. Calculations are only performed within each fraction-cohort combination; ns not significant (p≥0.05), * p<0.05, ** p<0.01, *** p<0.001, **** p<0.0001. Bottom: fold changes of the mean values compared with the controls in each fraction-antibody combination.

**Supplementary Figure 16.** Scatter-plots of tryptic phospo-peptides for both cohorts. Data is grouped according to fraction-cohort combination; antibody used for cohort comparison was HT7. TBS fractions are indicated blue and SI fractions pink. Top: box/scatter plots with medians, inter-quartile intervals and min/max intervals indicated. Middle: significances according to one-way ANOVA not assuming homoscedasticity (Brown-Forsythe/Welch) followed by unpaired t-test with Welch’s correction. Calculations are only performed within each fraction-antibody combination; ns not significant (p≥0.05), * p<0.05, ** p<0.01, *** p<0.001, **** p<0.0001. Bottom: fold changes of the mean values compared with the controls in each fraction-antibody combination.

**Supplementary Figure 17.** Comparison of the relative tryptic phospho-peptide abundance along the tau protein chain for IP with HT7 for both cohorts. The top panel represents the TBS fraction and the bottom panel the SI fraction. The profiles are the mean values for each cohort-disease combination.


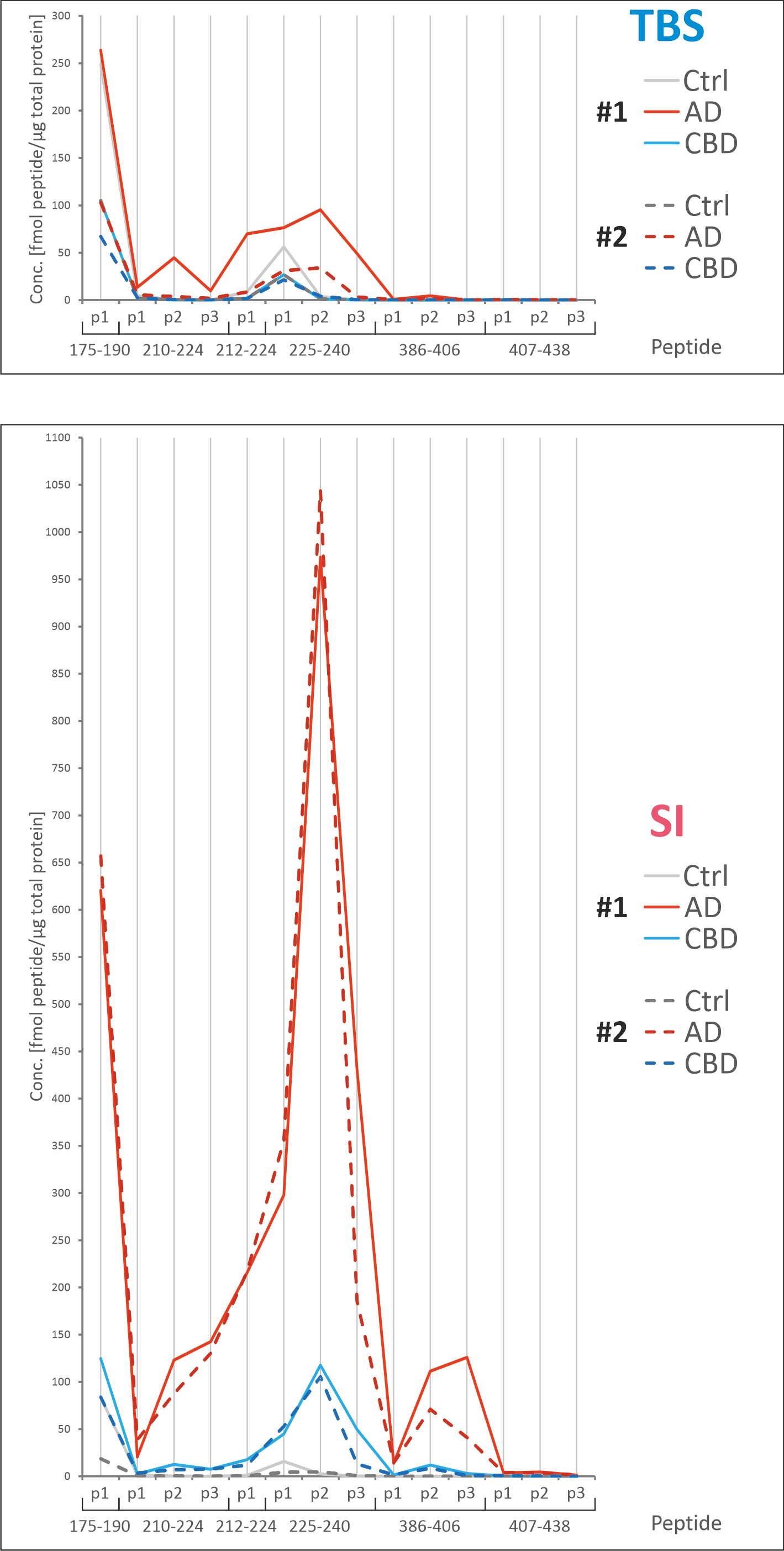


**Supplementary Figure 18.** Comparison of selected correlations for both cohorts and both the TBS and SI fractions immunoprecipitated with HT7. Correlations are Pearson’s r. Indicated with orange boxes are the high correlation with the respective disease-associated major isoform with a non-specific MTBR peptide for the SI fraction. Purple boxes indicate the high positive correlation with MTBR peptides and phosphorylated peptides in the proline-rich mid-region in the SI fraction. In AD, all MTBR peptides correlate with each other and with the phosphorylated peptides. In and CBD only the 4R specific and the non-specific peptides correlate. Correlation coefficient values are indicated by the colour scale and significance by size of the circles.


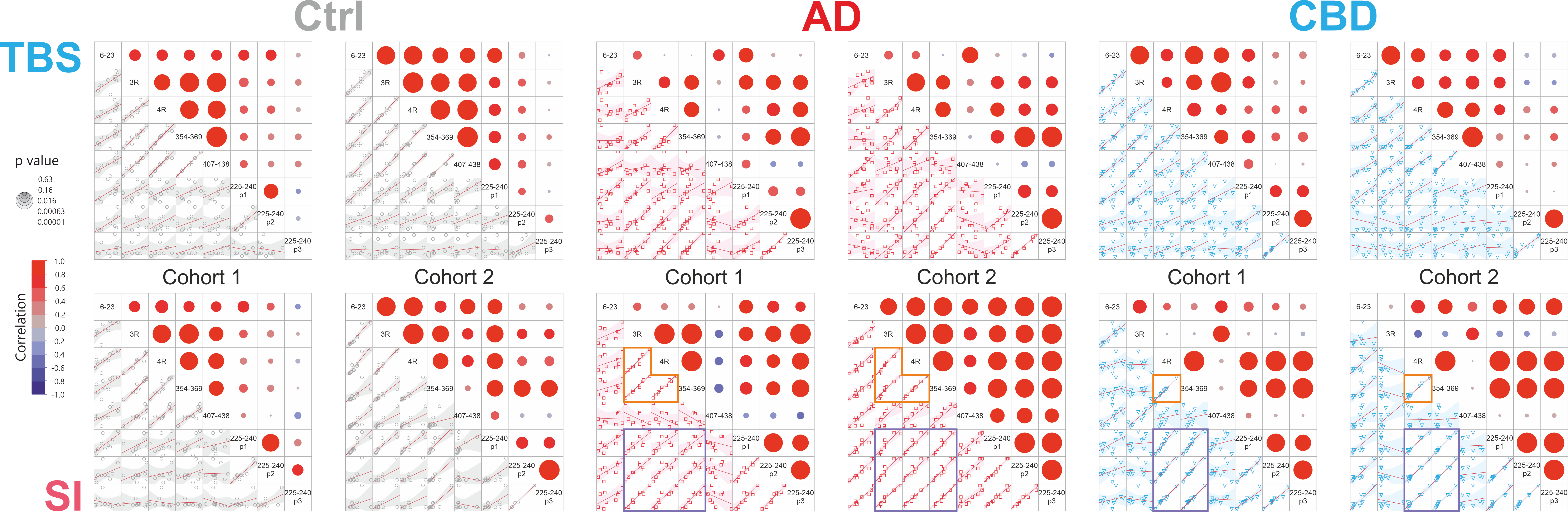


**Supplementary Figure 19 (separate Excel-file).** Comparison of correlations for both cohorts and both the TBS and SI fractions immunoprecipitated with HT7. Correlations between non-phosphorylated (top), non-phosphorylated and phosphorylated (bottom left), and phosphorylated (bottom right) tryptic peptides for each patient group. The data is divided by extraction fraction and peptide (N-terminal to C-terminal). Red indicates +1 positive correlation, white 0 no correlation, and blue -1 negative correlation. (a) controls, (b) AD, and (c) CBD.
