# Supplementary material for: Tau protein profiling in tauopathies: a human brain study
Source: Mol Neurodegener. 2024 Jul 19;19:54. doi: 10.1186/s13024-024-00741-9 (PMC11264707; doi:10.1186/s13024-024-00741-9)
Supplement: Supplementary file 6 — Supplementary Material 6: Information [file 13024_2024_741_MOESM6_ESM.docx]

**Tau protein profiling in tauopathies: a human brain study**

Juan Lantero-Rodriguez^1,2^*, Elena Camporesi^1,2^*, Laia Montoliu-Gaya^1,2^, Johan Gobom^1,2^, Diana Piotrowska^1,2^, Maria Olsson^1^, Irena Matečko Burmann^1,2,4^, Bruno Becker^1,2^, Ann Brinkmalm^1,2^, Björn M. Burmann^3,4^, Michael Perkinton^5^, Nicholas J. Ashton^1,2,6,7,8^, Nick C. Fox^9,10^, Tammaryn Lashley^9^, Henrik Zetterberg^1,2,9,10,11,12^, Kaj Blennow^1,2$^, Gunnar Brinkmalm^1,2$^

^1^ Department of Psychiatry & Neurochemistry, Institute of Neuroscience and Physiology, the Sahlgrenska Academy at the University of Gothenburg, Mölndal, Sweden

^2^ Clinical Neurochemistry Laboratory, Sahlgrenska University Hospital, Mölndal, Sweden

^3^ Department of Chemistry and Molecular Biology, University of Gothenburg, Gothenburg, Sweden

^4^ Wallenberg Centre for Molecular and Translational Medicine, University of Gothenburg, Gothenburg, Sweden

^5^ AstraZeneca Neuroscience Innovative Medicines, MedImmune Ltd, Cambridge CB21 6GH, UK

^6^ Centre for Age-Related Medicine, Stavanger University Hospital, Stavanger, Norway

^7^ Department of Old Age Psychiatry, Maurice Wohl Clinical Neuroscience Institute, King’s College London, London, UK

^8^ NIHR Biomedical Research Centre for Mental Health & Biomedical Research Unit for Dementia at South London & Maudsley NHS Foundation, London, UK

^9^ Department of Neurodegenerative Disease, Queen Square Institute of Neurology, University College London, London, UK

^10^ UK Dementia Research Institute, University College London, London, UK

^11^ Hong Kong Center for Neurodegenerative Diseases, Hong Kong, China

^12^ Wisconsin Alzheimer’s Disease Research Center, University of Wisconsin School of Medicine and Public Health, University of Wisconsin-Madison, Madison, WI, USA

*Contributed equally as first authors

^$^Contributed equally as senior authors

Corresponding author: Gunnar Brinkmalm, PhD. Department of Psychiatry and Neurochemistry, Institute of Neuroscience and Physiology, The Sahlgrenska Academy, University of Gothenburg, Gothenburg, Sweden.

Email: gunnar.brinkmalm@neuro.gu.se

**Supplementary information**

**1. Protein constructs**

Tau isoforms 0N3R, 1N4R and 2N4R cDNA were synthesized using GeneArt Custom DNA Fragment (Thermo Fisher Scientific) and PCR amplified with primers representing tau 5’ and 3’ sequence. PCR fragments were cloned directly into pET_SUMO, a plasmid for protein expression with N-terminal His-SUMO tag (Thermo Fisher Scientific). Clones with tau 0N3R or 1N4R insert were sequenced to verify the SUMO-tau fusion protein.

**2. Protein expression and purification**

*E. coli* BL21( λDE3) Star (Novagen) cells were transformed with a modified pET_Sumo plasmid (Invitrogen) harboring 2N4R, 1N4R, and 0N3R. [*U*–^15^N] isotope enriched proteins were produced using 2×M9 minimal medium [1] supplemented with (^15^NH_4_)Cl (Merck) (1 g/L) and *D*-glucose (2 mg/L) (Merck) as the sole nitrogen and carbon sources. The transformed cells were grown at 37 °C in medium supplemented with 50 µg/mL kanamycin until an OD_600_ ≈ 0.8 was reached. Expression was induced by addition of 1 mM isopropyl-thiogalactoside (IPTG) (Thermo Fisher Scientific) for 16 h at 22 °C. Cells were harvested by centrifugation, resuspended in a lysis buffer (20 mM NaPi, 500 mM NaCl, 10 mM MgCl_2_, pH=7.8), shock frozen in a liquid nitrogen and stored at -20 °C till usage. Protein purification was performed as described before [2]. Shortly, cells were lysed by four passes through an Emulsiflex C3 (Avestin) homogenizer and the cleared lysate were purified with HisTrap HP column (Cytivia). Fractions containing tau proteins were pooled and dialyzed against human SenP1 cleavage buffer. Sumo-tag was cleaved with SenP1 protease (Addgene #16356) [3] and further purification was done by a second HisTrap HP column step. Fractions containing cleaved Tau protein in the flow-through were collected, concentrated, and subsequently purified by gel filtration using a HiLoad Superdex 10/60 200 pg preparative SEC columns (Cytivia) pre-equilibrated PBS buffer. The pure [*U*–^15^N] tau fractions were concentrated to about 1 mg/mL, flash frozen in liquid nitrogen, and stored at -80 °C till usage.

**3. Titration of labelled protein standards**

Concentrated stock solutions (70-216 µM in ammonium acetate) of [*U*–^15^N]-labelled recombinant tau protein isoforms were stepwise diluted in ammonium bicarbonate to 0.2 µM and stored at -80 °C until usage. The [*U*–^15^N]-labelled tau isoforms 0N3R, 1N4R, and 2N4R were then added at nominally equal amounts to separate aliquots of identical TBS pool samples, which were then IP’d using Tau12 antibody and subsequently digested using trypsin. Each combination was analysed in triplicate. For seven tryptic peptides common to all isoforms the ratio, *R_H/L_*, of the peak area of the isotope-labelled peptide, *A_H_*, to that of the peptide from brain tau, *A_L_*, was determined for each isoform (1a-c) and mean values and CVs calculated.

|  | $R_{H/L}(0N3R)=\frac{A_{H}(0N3R)}{A_{L}(0N3R)}$ | (1a) |
| --- | --- | --- |
|  | $R_{H/L}(1N4R)=\frac{A_{H}(1N4R)}{A_{L}(1N4R)}$ | (1b) |
|  | $R_{H/L}(2N4R)=\frac{A_{H}(2N4R)}{A_{L}(2N4R)}$ | (1c) |

In the next step, the 1N4R standard was chosen as the reference and second ratios of the seven peptide ratios from the 0N3R and 2N4R standards to the 1N4R standard were determined (2a-b).

|  | $R_{0N3R/1N4R}=\frac{R_{H/L}(0N3R)}{R_{H/L}(1N4R)}$ | (2a) |
| --- | --- | --- |
|  | $R_{2N4R/1N4R}=\frac{R_{H/L}(2N4R)}{R_{H/L}(1N4R)}$ | (2b) |

From these second ratios (which should theoretically be the same for any set of two standards) mean values and CVs (<15%) were determined. Mean value for *R*_0N3R/1N4R_ peptide ratios was 0.72 and for *R*_2N4R/1N4R_ 0.91. Thus, the final equimolar mixture of 0N3R/1N4R/2N4R was set to 1.4/1.0/1.1 (v/v/v) and the respective isoforms were each added at a *nominal* amount 133 fmol per sample. From the CVs the accuracy could be estimated to be of the order of 15-20%. However, measured *differences* within this study were more precise since the same mix was used in all experiments.

**4. Sample randomisation**

For Cohort 1, the analysis order of all samples was randomised in a two-step way to get the different patient groups spread out evenly throughout each four-day acquisition occasion. First, all samples were randomised within the patient group. The first sample in each group was then sorted into another group – mixed group one. Then the second sample in each patient group was sorted into mixed group two, and so on. The eleventh PSP sample was then randomly added to one of the ten mixed groups. Second, each of these groups was then randomised once more to complete the two-step random process. The six QC samples and nine blank injections were then inserted to the analysis sequence so that they were evenly spread out. For Cohort 2 the same procedure was applied but adapted to the particular sample set.

**5. Database search settings**

Both Mascot (Matrix Science) and PEAKS Studio (Bioinformatics Solutions) were used for database searches. Mascot Daemon v2.6.1 was used to initiate the processing. Charge and isotope deconvolution of mass spectra was performed using Mascot Distiller v2.6.3 before submitting searches to Mascot database search software v2.6.1. Similarly PEAKS Studio Xpro has a built-in charge and isotope deconvolution feature that was employed before performing the database search. Searches were made against both SwissProt/Uniprot isoforms and a custom made human tau-only database containing all tau isoforms.

Mascot Distiller processing parameters as well as the Mascot database search parameters were the same as described in [4], except only collision-induced dissociation (CID) type fragment spectra were searched and that for the tau-only database search the variable PTMs were: Acetyl (Protein N-term), Oxidation (M), Phospho (ST), Label 13C(6) 15N(4) (R), Label 13C(6) 15N(2) (K), while for SwissProt/Uniprot isoforms variable PTMs were: Oxidation (M). PEAKS Studio search parameters were: Create Project options: [Instrument type = Orbitrap (Orbi-Orbi); Fragment = HCD; Acquisition = DDA]. Data Refinement options: [Merge Scans = not enabled; Correct Precursor = enabled; Mass only (recommended) = enabled; Filter Scans = not enabled]; Associate feature with chimera scan = not enabled; Filter Features = not enabled. Identification options: [Error Tolerance: Precursor mass = 20 ppm using monoisotopic mass; Fragment ion = 0.05 Da; Enzyme = None; Digest Mode = Unspecific; Selected Fixed PTM = none; Selected Variable PTM = Acetylation (Protein N-term), Oxidation (M), Phospho (STY), 13C(6) 15N(4) Silac label, 13C(6) 15N(2) Silac label; max variable PTM per peptide = 6; Database = custom made tau_isoforms_human, Uniprot_SwissProt_isoforms]. In a separate search the [*U*–^15^N]-protein standards were addressed: Selected Fixed PTM = SILAS 15N(1), SILAS 15N(2), SILAS 15N(3), SILAC [sic!] 15N(4); Selected Variable PTM = Oxidation (M); max variable PTM per peptide = 3. Apart from the PTMs all other parameters were the same.

**6. Calculation of peptide concentrations**

With the approach outlined above for addition of isotope-labelled protein standards, the procedure to calculate brain peptide concentrations was as follows. Each of the three protein isoforms were added at a nominal amount of 133 fmol per sample. This means that for all tryptic peptides common to all isoforms the added amount corresponded to 3×133 = 400 fmol (since they had all three protein isoforms as sources). A light-to-heavy ratio of 1 then corresponded to 400 fmol for common peptides in that sample. Isoform dependent isotope-labelled peptides were instead present at 133 fmol for the 0N, 1N, 2N, and 3R specific peptides (which only had one source of protein standard) and at 267 fmol for the 4R specific peptide (which had two sources of protein standards, 1N4R and 2N4R). Hence, for these isotope-specific peptides, a light-to-heavy ratio of 1 corresponded to 133 fmol or 267 fmol, respectively.

Phosphorylated isotope-labelled peptides were each added at a nominal (as stated by the provider) amount of 100 fmol per sample. Since an explorative approach was used, quantification was performed on the intact peptide. Some peptides only differed by the position of the phosphate group, which did not affect retention time noticeably. Therefore, for such phospho-peptides, separation of chromatographic peak areas was not possible and this had to be taken into account when calculating the concentration. The involved peptides were 195-209-p1 (phosphorylation at Ser-202 or Thr-205) and 386-406-p1 (phosphorylation at Ser-396 or Ser-404), which together were added at 200 fmol per sample, and 225-240-p2 (phosphorylation at Thr-231 and either of Ser-235, Ser-237, or Ser-238) and 225-240-p3 (phosphorylation at Thr-231 and either pair of Ser-235+Ser-237, Ser-235+Ser-238, or Ser-237+Ser-238), which together were added at 300 fmol per sample.

Since the quantitative method was optimised using mainly TBS fraction material, some phosphorylated peptides later found in SI fraction were not observed and investigated until after spectra from all samples were acquired. For these different surrogate standards were used, since isotope-labelled peptides were missing. For 210-224-p3 the labelled 210-224-p2 peak area was used, for 386-406-p3 the labelled 386-406-p2 area was used, and for the three 407-438-p1, -p2, -p3 the [*U*–^15^N]-labelled non-phosphorylated 407-438 area was used.

Since the actual peptide amount may vary substantially from the amount stated by the manufacturer it is typically not possible to compare the amounts between different peptides. To do this, protein standards are required. Thus, to assess the amount of phosphorylated peptides along the protein chain, the mean area of several tryptic peptides from the [*U*–^15^N]-protein standards were used as surrogate. This approach introduces other variations due to, for example, ionisation efficiency of the brain derived peptides or time dependent spray variation since the elution time is no longer the same. However, since we particularly wanted to compare the same peptides with different degree of phosphorylation, this approach was deemed to be more favourable than using standards less well defined. Naïvely one might assume that the number of phosphate groups would negatively affect the ionisation efficiency. This is, however, not the case; the effect of phosphate on ionization efficiency is more complicated and no general assumption can be made [5, 6]. In summary, quantitative comparisons of different peptides performed in this way should be interpreted with caution.

**7. Harmonisation of data acquisitions**

For Cohort 1, data was collected at seven occasions spread out during several months. Therefore, QC samples were used to adjust for variations that could occur, for example, potential time dependent drifts during the four-day acquisition of one fraction-antibody combination or between acquisition occasions. QC samples were represented by the same TBS pool and were at each occasion IP’d with Tau12 antibody to produce nominally identical data throughout the whole study. Within an acquisition occasion there was no drift observed; however, between occasions there were noticeable differences. The median ratios for the non-phosphorylated peptides that had [*U*–^15^N]-protein standards varied between 0.8 and 1.6 compared with the first occasion, and for the phosphorylated peptides the ratios varied between 0.8 and 2.1. The reason for this is unclear, but several parameters (*e.g.*, storage time, general sample handling, IP efficiency, digestion, instrument condition) can contribute to the total variation.

Harmonisation between the occasions was performed on an individual peptide level. The median ratio for the six QC samples analysed at a particular occasion was calculated for each peptide analysed. The initial occasion Tau12/TBS was set as reference. For each subsequent occasion and peptide, the value obtained was divided by that of the initial occasion and a harmonisation ratio was obtained. All peptide ratios obtained from the study samples were then divided by the corresponding harmonisation ratio.

For Cohort 2, the HT7 antibody was used also for the 11 QCs; otherwise harmonisation was implemented as described above.

**8. References**

1. Azatian, S.B., N. Kaur, and M.P. Latham, *Increasing the buffering capacity of minimal media leads to higher protein yield.* J Biomol NMR, 2019. **73**(1-2): p. 11–17.

2. Lesovoy, D.M., et al., *Unambiguous Tracking of Protein Phosphorylation by Fast High-Resolution FOSY NMR*.* Angew Chem Int Ed Engl, 2021.

3. Mikolajczyk, J., et al., *Small ubiquitin-related modifier (SUMO)-specific proteases: profiling the specificities and activities of human SENPs.* J Biol Chem, 2007. **282**(36): p. 26217–24.

4. Brinkmalm, G., et al., *An online nano-LC-ESI-FTICR-MS method for comprehensive characterization of endogenous fragments from amyloid beta and amyloid precursor protein in human and cat cerebrospinal fluid.* J Mass Spectrom, 2012. **47**(5): p. 591-603

5. Gao, Y. and Y. Wang, *A method to determine the ionization efficiency change of peptides caused by phosphorylation.* J Am Soc Mass Spectrom, 2007. **18**(11): p. 1973-6.

6. Steen, H., et al., *Phosphorylation analysis by mass spectrometry: myths, facts, and the consequences for qualitative and quantitative measurements.* Mol Cell Proteomics, 2006. **5**(1): p. 172-81.
