# Supplementary material for: Tau protein profiling in tauopathies: a human brain study
Source: Mol Neurodegener. 2024 Jul 19;19:54. doi: 10.1186/s13024-024-00741-9 (PMC11264707; doi:10.1186/s13024-024-00741-9)
Supplement: Supplementary file 8 — Supplementary Material 8: Tables [file 13024_2024_741_MOESM8_ESM.docx]

**Supplementary Table 1.** Full case demographics of the two tauopathies cohorts from frontal grey matter. The ABC score is a composite of three different assessments incorporating, (A) Thal phases of amyloid deposition, (B) Braak stage of NFTs and (C) score of amyloid neuritic plaques (CERAD). AAO = age at onset, AAD = age at death, PM delay = post-mortem delay, CERAD = consortium to establish a registry for Alzheimer´s disease, CAA = cerebral amyloid angiopathy, na = not analyzed.

| **Cohort 1** | | | | | | | | | | | |
| --- | --- | --- | --- | --- | --- | --- | --- | --- | --- | --- | --- |
| **Diagnosis** | **Gender** | **AAO (years)** | **AAD (years)** | **PM delay (h)** | **Brain mass (g)** | **ApoE genotype** | **CAA** | **Thal phase** | **Braak staging** | **CERAD score** | **ABC score** |
| **Ctrl** | M | na | 101 | 60.25 | 1450 | ε2/ε3 | 1 | 0 | I | 0 | A0B1C0 |
|  | M | na | 38 | 80.35 | 1581 | ε3/ε4 | 0 | 0 | 0 | 0 | A0B0C0 |
|  | F | na | 86 | 119.05 | 1230 | ε3/ε4 | 0 | 4 | I | 1 | A3B1C1 |
|  | F | na | 87 | 51.40 | 1114 | ε3/ε3 | 0 | 1 | I | 0 | A1B1C1 |
|  | F | na | 86 | 40.20 | 1238 | ε3/ε3 | 0 | 0 | 0 | 0 | A0B0C0 |
|  | F | na | 78 | 29.30 | 1225 | ε2/ε2 | 0 | 1 | I | 0 | A0B1C0 |
|  | F | na | 68 | 45.05 | 1330 | ε2/ε3 | 0 | 0 | 0 | 0 | A0B0C0 |
|  | M | na | 69 | 171.00 | 1435 | ε3/ε3 | 1 | 3 | I | 1 | A2B1C1 |
|  | F | na | 79 | 88.50 | 1288 | ε3/ε3 | 0 | 2 | I | 1 | A2B1C1 |
|  | M | na | 95 | 89.40 | 1346 | ε2/ε3 | 0 | 2 | I | 1 | A2B1C1 |
| **AD** | M | 54 | 65 | 34.25 | 1089 | ε4/ε4 | 3 | 5 | VI | 3 | A3B3C3 |
|  | M | 48 | 63 | 31.42 | 1042 | ε3/ε3 | 3 | 5 | VI | 3 | A3B3C3 |
|  | M | 44 | 59 | 90.45 | 1338 | ε3/ε3 | 3 | 5 | VI | 3 | A3B3C3 |
|  | F | 69 | 76 | 42.00 | 1236 | ε3/ε4 | 0 | 5 | VI | 2 | A3B3C2 |
|  | M | 71 | 86 | 95.10 | 1203 | ε3/ε3 | 3 | 5 | V | 3 | A3B3C3 |
|  | M | 54 | 67 | 32.15 | 1458 | ε4/ε4 | 3 | 5 | VI | 3 | A3B3C3 |
|  | F | 49 | 69 | 40.10 | 986 | ε4/ε4 | 3 | 5 | VI | 2 | A3B3C2 |
|  | M | 52 | 71 | 45.35 | 1097 | ε3/ε3 | 3 | 5 | VI | 3 | A3B3C3 |
|  | F | 58 | 62 | 92.20 | 1234 | ε3/ε4 | 1 | 5 | VI | 2 | A3B3C2 |
|  | F | 57 | 76 | 57.50 | 1303 | ε3/ε4 | 2 | 5 | VI | 2 | A3B3C2 |
| **PSP** | M | 73 | 84 | 66.34 | 1271 | na | na | na | na | na | na |
|  | F | 66 | 79 | 73.55 | 1141 | na | na | na | na | na | na |
|  | M | 57 | 62 | 72.20 | 1369 | na | na | na | na | na | na |
|  | M | 76 | 84 | 50.00 | 1370 | na | na | na | na | na | na |
|  | F | 75 | 84 | 70.00 | 1095 | na | na | na | na | na | na |
|  | M | 71 | 83 | 32.35 | 1137 | na | na | na | na | na | na |
|  | F | 60 | 68 | 36.50 | 1177 | na | na | na | na | na | na |
|  | F | 84 | 92 | 43.10 | 1118 | na | na | na | na | na | na |
|  | F | 67 | 77 | 30.42 | 1095 | na | na | na | na | na | na |
|  | M | 78 | 88 | 48.45 | 1307 | na | na | na | na | na | na |
|  | M | 63 | 71 | 4.35 | 1179 | na | na | na | na | na | na |
| **CBD** | M | 63 | 69 | 81.36 | 1291 | na | na | na | na | na | na |
|  | M | 56 | 62 | 45.15 | 1173 | na | na | na | na | na | na |
|  | F | 58 | 69 | 103.15 | 917 | na | na | na | na | na | na |
|  | M | 54 | 61 | 102.30 | 1389 | na | na | na | na | na | na |
|  | M | 58 | 65 | 48.04 | 1232 | na | na | na | na | na | na |
|  | M | 69 | 77 | 37.00 | 1113 | na | na | na | na | na | na |
|  | F | 62 | 68 | 98.25 | 1172 | na | na | na | na | na | na |
|  | M | 67 | 73 | 54.25 | 1154 | na | na | na | na | na | na |
|  | M | 57 | 72 | 84.10 | 1378 | na | na | na | na | na | na |
|  | F | 66 | 70 | 75.00 | 1050 | na | na | na | na | na | na |
| **PiD** | F | 60 | 71 | 87.15 | 849 | na | na | na | na | na | na |
|  | M | 52 | 67 | 30.30 | 982 | na | na | na | na | na | na |
|  | M | 57 | 62 | 24.00 | 1166 | na | na | na | na | na | na |
|  | M | 70 | 78 | 74.45 | 1280 | na | na | na | na | na | na |
|  | M | 53 | 67 | 73.05 | 1000 | na | na | na | na | na | na |
|  | M | 51 | 68 | 79.50 | 854 | na | na | na | na | na | na |
|  | M | 63 | 75 | 46.30 | 933 | na | na | na | na | na | na |
|  | M | 60 | 68 | 94.45 | 1209 | na | na | na | na | na | na |
|  | M | 55 | 72 | 97.35 | 805 | na | na | na | na | na | na |
|  | M | 54 | 64 | 43.30 | 1040 | na | na | na | na | na | na |
| **Cohort 2** | | | | | | | | | | | |
| **Diagnosis** | **Gender** | **AAO (years)** | **AAD (years)** | **PM delay (h)** | **Brain mass (g)** | **ApoE genotype** | **CAA** | **Thal phase** | **Braak staging** | **CERAD score** | **ABC score** |
| **Ctrl** | M | na | 84 | 79.17 | 1468 | ε3/ε3 | 0 | 0 | 0 | 0 | A0B0C0 |
|  | F | na | 83 | 53.58 | 1283 | ε3/ε3 | 1 | 3 | II | 1 | A2B1C1 |
|  | M | na | 83 | 105.47 | 1244 | ε3/ε3 | 2 | 3 | IV | 2 | A2B2C2 |
|  | M | na | 76 | 79.00 | 1366 | ε3/ε4 | 0 | 1 | II | 0 | A1B1C0 |
|  | M | na | 88 | 27.50 | 1330 | ε3/ε3 | 3 | 3 | IV | 2 | A2B2C2 |
|  | M | na | 79 | 3.50 | 1355 | ε3/ε3 | 0 | 0 | II | 0 | A0B1C0 |
|  | F | na | 77 | 30.23 | 1531 | ε3/ε3 | 0 | 3 | I | 1 | A2B1C1 |
|  | M | na | 73 | 54.33 | 1498 | ε2/ε4 | 3 | 4 | III | 2 | A3B2C2 |
|  | M | na | 64 | 80.00 | 1695 | ε3/ε3 | 0 | 1 | 0 | 0 | A1B0C0 |
|  | M | na | 73 | 47.00 | 1291 | ε3/ε3 | 0 | 1 | IV | 0 | A0B1C0 |
| **AD** | M | 59 | 68 | 85.42 | 1381 | ε3/ε4 | 2 | 5 | VI | 3 | A3B3C3 |
|  | F | 59 | 79 | 30.42 | 961 | ε3/ε3 | 3 | 5 | VI | 3 | A3B3C3 |
|  | F | 51 | 67 | 49.75 | 965 | ε3/ε3 | 2 | 5 | VI | 3 | A3B3C3 |
|  | M | 76 | 82 | 24.00 | 1208 | ε3/ε4 | 3 | 5 | VI | 2 | A3B3C2 |
|  | F | 74 | 87 | 53.50 | 1065 | ε3/ε4 | 3 | 5 | VI | 3 | A3B3C3 |
|  | F | 53 | 72 | 56.50 | 1190 | ε3/ε4 | 1 | 5 | VI | 3 | A3B3C3 |
|  | F | 54 | 71 | 76.75 | 973.5 | na | 3 | 5 | VI | 3 | A3B3C3 |
|  | M | 54 | 74 | 42.50 | 1212 | na | 1 | 5 | VI | 3 | A3B3C3 |
|  | F | 63 | 75 | 75.50 | 1124 | na | 3 | 5 | VI | 3 | A3B3C3 |
|  | M | 73 | 83 | 92.08 | 1140 | na | 3 | 5 | VI | 2 | A3B3C2 |
| **CBD** | F | 62 | 69 | 80.80 | 1100 | na | 0 | 0 | II | 0 | A0B1C0 |
|  | F | 58 | 69 | 103.25 | 917 | na | 0 | 0 | 0 | 0 | A0B0C0 |
|  | M | 57 | 64 | 41.42 | 1456 | na | 0 | 0 | 0 | 0 | A0B0C0 |
|  | M | 61 | 66 | 122.08 | 1449 | na | 0 | 0 | 0 | 0 | A0B0C0 |
|  | M | 60 | 65 | 50.75 | 1460 | na | 0 | 2 | II | 0 | A1B1C0 |
|  | F | 72 | 83 | 85.67 | 932 | na | 1 | 5 | IV | 2 | A3B2C2 |
|  | F | 74 | 82 | 42.92 | 1102 | na | 1 | 5 | III | 1 | A3B2C1 |
|  | F | 60 | 71 | 103.50 | 1312 | na | 0 | 0 | II | 0 | A0B1C0 |
|  | M | 70 | 77 | 54.00 | 1208 | na | 2 | 5 | II | 2 | A3B1C2 |
|  | F | 57 | 64 | 95.58 | 1141 | na | 0 | 1 | II | 0 | A1B1C0 |

**Supplementary Table 2.** Characteristics of quantified peptides.

| **Sequence** | **Isoform** | **Peptide** | **#phosphates** | **Mass [Da]** | **m/z** | **Charge** | **NCE^b^** | **RT [min]^c^** | **Label** |
| --- | --- | --- | --- | --- | --- | --- | --- | --- | --- |
| QEFEVMEDHAGTYGLGDR |  | 6-23 |  | 2052.8847 | 685.3022 | 3 | 26 | 34 | [*U*-^15^N] |
| DQGGYTMHQDQEGDTDAGLK |  | 25-44 |  | 2164.8967 | 722.6395 | 3 | 18 | 23 | [*U*-^15^N] |
| AEEAGIGDTPSLEDEAAGHVTQAR^a^ | 0N | 45-68 |  | 2423.1201 | 808.7140 | 3 | 28 | 32 | [*U*-^15^N] |
| STPTAEAEEAGIGDTPSLEDEAAGHVTQAR | 1N | 68-97 |  | 3009.3799 | 1004.1339 | 3 | 26 | 35 | [*U*-^15^N] |
| STPTAEDVTAPLVDEGAPGK | 2N | 68-87 |  | 1953.9531 | 977.9838 | 2 | 20 | 35 | [*U*-^15^N] |
| SGYSSPGSPGTPGSR |  | 195-209 |  | 1392.6270 | 697.3208 | 2 | 20 | 20 | [*U*-^15^N] |
| TPSLPTPPTR |  | 212-221 |  | 1065.5819 | 533.7982 | 2 | 20 | 27 | [*U*-^15^N] |
| LQTAPVPMPDLK |  | 243-254 |  | 1308.7112 | 655.3629 | 2 | 22 | 36 | [*U*-^15^N] |
| VQIVYKPVDLSK^a^ | 3R | 275-286 |  | 1387.8075 | 463.6098 | 3 | 26 | 29 | [*U*-^15^N] |
| HVPGGGSVQIVYKPVDLSK | 4R | 299-317 |  | 1979.0840 | 660.7019 | 3 | 28 | 31 | [*U*-^15^N] |
| IGSLDNITHVPGGGNK |  | 354-369 |  | 1577.8162 | 526.9460 | 3 | 25 | 28 | [*U*-^15^N] |
| HLSNVSSTGSIDMVDSPQLATLADEVSASLAK |  | 407-438 |  | 3242.5977 | 1081.8732 | 3 | 25 | 54 | [*U*-^15^N] |
| TPPAPKTPPSSGEPPK |  | 175-190 | p1 | 1666.7968 | 556.6062 | 3 | 25 | 21 | ^13^C_6_,^15^N_2_-Lys |
| SGYSSPGSPGTPGSR |  | 195-209 | p1 | 1472.5933 | 737.3039 | 2 | 25 | 22 | ^13^C_6_,^15^N_4_-Arg |
| SGYSSPGSPGTPGSR |  | 195-209 | p2 | 1552.5596 | 777.2871 | 2 | 25 | 24 | ^13^C_6_,^15^N_4_-Arg |
| SRTPSLPTPPTREPK |  | 210-224 | p1 | 1742.8717 | 581.9645 | 3 | 25 | 23 | ^13^C_6_,^15^N_2_-Lys |
| SRTPSLPTPPTREPK |  | 210-224 | p2 | 1822.8380 | 608.6199 | 3 | 25 | 27 | ^13^C_6_,^15^N_2_-Lys |
| SRTPSLPTPPTREPK |  | 210-224 | p3 | 1902.8043 | 635.2754 | 3 | 25 | 31 | ^13^C_6_,^15^N_2_-Lys |
| TPSLPTPPTREPK |  | 212-224 | p1 | 1499.7385 | 500.9201 | 3 | 25 | 28 | ^13^C_6_,^15^N_2_-Lys |
| KVAVVRTPPKSPSSAK |  | 225-240 | p1 | 1730.9444 | 577.9888 | 3 | 25 | 18 | ^13^C_6_,^15^N_2_-Lys |
| KVAVVRTPPKSPSSAK |  | 225-240 | p2 | 1810.9108 | 604.6442 | 3 | 25 | 19 | ^13^C_6_,^15^N_2_-Lys |
| KVAVVRTPPKSPSSAK |  | 225-240 | p3 | 1890.8771 | 631.2996 | 3 | 25 | 21 | ^13^C_6_,^15^N_2_-Lys |
| TDHGAEIVYKSPVVSGDTSPR |  | 386-406 | p1 | 2294.0580 | 765.6933 | 3 | 25 | 27 | ^13^C_6_,^15^N_4_-Arg |
| TDHGAEIVYKSPVVSGDTSPR |  | 386-406 | p2 | 2374.0243 | 792.3487 | 3 | 25 | 29 | ^13^C_6_,^15^N_4_-Arg |
| TDHGAEIVYKSPVVSGDTSPR |  | 386-406 | p3 | 2453.9907 | 819.0042 | 3 | 25 | 35 | ^13^C_6_,^15^N_4_-Arg |
| HLSNVSSTGSIDMVDSPQLATLADEVSASLAK |  | 407-438 | p1 | 3322.5640 | 1108.5286 | 3 | 25 | 59 | [*U*-^15^N] |
| HLSNVSSTGSIDMVDSPQLATLADEVSASLAK |  | 407-438 | p2 | 3402.5303 | 1135.1840 | 3 | 25 | 62 | [*U*-^15^N] |
| HLSNVSSTGSIDMVDSPQLATLADEVSASLAK |  | 407-438 | p3 | 3482.4966 | 1161.8395 | 3 | 25 | 69 | [*U*-^15^N] |

^a^ Only tryptic (or LysC produced) peptide is isoform specific; *i.e.*, for 0N and 3R the preceeding amino acid is Lys. No indication of endogenous cleavage at these sites have been observed.

^b^ NCE = normalised collision energy. For peptides not included in this list the value was set to 25.

^c^ The retention time varied less than ±0.5 min within an occasion and typically up to ±3 min between occasions.

**Supplementary Table 3 (separate Excel-file).** Ratio data from the measured peak areas from the linearity test for all peptides with a labelled standard shown in Suppl. Figure 3. Different amounts of pooled AD sample was immuprecipitated using HT7, while keeping the amount of isotope-labelled standards constant. Fitting was performed on individual data using the Weighted Linear Regression Excel add-in tool by Real Statistics ([www.real-statistics.com](http://www.real-statistics.com)), where the weight was 1/x.

**Supplementary Table 4.** Relative isoform amounts for both cohorts and all fraction and antibody combinations. Values are mean (SD).

|  |  | **0N** | | **1N** | | **2N** | | **3R** | | **4R** | |
| --- | --- | --- | --- | --- | --- | --- | --- | --- | --- | --- | --- |
| **Cohort 1** | | TBS | SI | TBS | SI | TBS | SI | TBS | SI | TBS | SI |
| **Tau12** | Ctrl | 52.1 (12.8) | - | 45.3 (4.7) | - | 2.6 (0.7) | - | 63.7 (27.1) | - | 36.3 (17.0) | - |
| **HT7** | Ctrl | 53.2 (14.1) | 57.1 (8.8) | 44.7 (4.4) | 41.4 (2.8) | 2.1 (0.6) | 1.5 (0.5) | 59.4 (21.9) | 61.8 (15.0) | 40.6 (17.0) | 38.2 (11.1) |
| **77G7** | Ctrl | 57.1 (21.4) | 60.8 (16.4) | 39.7 (4.4) | 36.5 (3.5) | 3.1 (1.2) | 2.7 (0.8) | 52.2 (19.2) | 54.6 (17.2) | 47.8 (16.1) | 45.4 (13.0) |
| **TauAB** | Ctrl | 51.1 (29.6) | 52.5 (23.5) | 47.0 (6.5) | 45.4 (5.7) | 1.9 (1.0) | 2.2 (1.1) | 61.9 (29.6) | 59.8 (25.6) | 38.1 (18.8) | 40.2 (16.1) |
| **Tau12** | AD | 49.9 (18.6) | - | 46.0 (6.6) | - | 4.0 (2.4) | - | 65.3 (22.2) | - | 34.7 (11.9) | - |
| **HT7** | AD | 50.7 (15.0) | 68.8 (20.9) | 46.3 (8.4) | 29.1 (3.0) | 3.0 (1.6) | 2.1 (1.4) | 59.3 (18.7) | 63.4 (30.3) | 40.7 (12.4) | 36.6 (19.5) |
| **77G7** | AD | 58.6 (30.5) | 72.9 (23.1) | 37.1 (8.3) | 24.9 (1.7) | 4.3 (1.8) | 2.3 (1.1) | 49.6 (14.7) | 68.9 (48.8) | 50.4 (12.6) | 31.1 (25.0) |
| **TauAB** | AD | 52.8 (31.2) | 68.9 (22.5) | 45.1 (7.8) | 28.6 (2.8) | 2.1 (2.0) | 2.5 (1.5) | 62.9 (27.6) | 56.3 (32.7) | 37.1 (16.2) | 43.7 (34.6) |
| **Tau12** | PSP | 45.8 (13.3) | - | 50.8 (5.8) | - | 3.3 (1.1) | - | 64.1 (21.4) | - | 35.9 (13.9) | - |
| **HT7** | PSP | 48.4 (15.7) | 54.4 (16.4) | 49.1 (6.7) | 43.8 (4.4) | 2.5 (0.8) | 1.9 (0.6) | 52.2 (19.2) | 46.7 (19.5) | 47.8 (19.6) | 53.3 (30.4) |
| **77G7** | PSP | 52.7 (25.4) | 57.4 (29.4) | 43.4 (7.0) | 39.5 (8.6) | 3.9 (1.7) | 3.1 (1.4) | 50.1 (20.3) | 43.0 (20.3) | 49.9 (16.9) | 57.0 (21.7) |
| **TauAB** | PSP | 43.6 (28.5) | 54.6 (32.5) | 54.5 (10.7) | 43.2 (8.2) | 1.9 (1.5) | 2.2 (1.4) | 60.1 (37.9) | 36.0 (23.0) | 39.9 (22.0) | 64.0 (41.9) |
| **Tau12** | CBD | 51.7 (27.9) | - | 45.6 (12.3) | - | 2.7 (1.4) | - | 57.3 (38.0) | - | 42.7 (21.4) | - |
| **HT7** | CBD | 53.4 (31.2) | 61.3 (19.3) | 44.7 (10.6) | 37.1 (6.3) | 2.0 (1.0) | 1.6 (0.6) | 40.3 (25.1) | 16.7 (6.4) | 59.7 (27.5) | 83.3 (36.4) |
| **77G7** | CBD | 59.9 (38.9) | 67.6 (22.8) | 36.2 (7.5) | 29.9 (3.8) | 4.0 (1.7) | 2.6 (1.0) | 42.6 (24.7) | 13.1 (5.2) | 57.4 (28.2) | 86.9 (38.3) |
| **TauAB** | CBD | 51.4 (29.8) | 66.9 (25.3) | 47.2 (6.8) | 31.2 (4.1) | 1.4 (1.2) | 1.9 (0.8) | 30.7 (18.0) | 7.9 (2.8) | 69.3 (29.0) | 92.1 (44.5) |
| **Tau12** | PiD | 50.7 (18.0) | - | 46.0 (16.1) | - | 3.3 (2.2) | - | 64.0 (38.4) | - | 36.0 (18.9) | - |
| **HT7** | PiD | 55.1 (22.5) | 62.6 (17.5) | 42.5 (12.2) | 35.7 (7.7) | 2.4 (1.4) | 1.7 (1.4) | 58.1 (29.4) | 84.5 (60.2) | 41.9 (24.3) | 15.5 (6.9) |
| **77G7** | PiD | 65.6 (83.0) | 67.6 (19.0) | 30.6 (11.0) | 29.4 (6.9) | 3.8 (2.7) | 3.1 (1.8) | 45.7 (34.5) | 81.4 (55.0) | 54.3 (45.3) | 18.6 (8.6) |
| **TauAB** | PiD | 44.5 (36.4) | 65.4 (27.6) | 52.9 (19.9) | 32.6 (7.9) | 2.5 (4.0) | 2.0 (1.6) | 68.3 (65.8) | 87.6 (68.5) | 31.7 (25.0) | 12.4 (7.2) |
| **Cohort 2** | |  |  |  |  |  |  |  |  |  |  |
| **HT7** | Ctrl | 52.3 (14.1) | 56.1 (38.3) | 44.3 (11.2) | 41.0 (25.4) | 3.4 (1.3) | 2.8 (2.0) | 63.2 (17.2) | 59.8 (53.0) | 36.8 (13.6) | 40.2 (28.8) |
| **HT7** | AD | 51.4 (14.9) | 68.8 (49.0) | 44.9 (13.4) | 27.9 (23.7) | 3.8 (1.4) | 3.3 (3.2) | 61.4 (20.4) | 66.4 (69.2) | 38.6 (14.4) | 33.6 (27.9) |
| **HT7** | CBD | 50.9 (9.5) | 68.2 (43.1) | 46.2 (9.4) | 29.5 (8.1) | 3.0 (0.6) | 2.4 (0.9) | 57.8 (17.1) | 8.9 (9.3) | 42.2 (11.2) | 91.1 (78.5) |

**Supplementary Table 5**. Identified phospho-peptides. For each patient group and fraction-antibody combination the number of fragment ion spectra are given where the respective compound was identified as determined by PEAKS Studio Xpro. Many of the identifications were confirmed manually; however, the precise phosphate positions in several of the less abundant peptides, particularly the multiply phosphorylated ones, should be interpreted with caution. White background indicates compound not acquired.
